# Targeted Next-Generation Sequencing Indicates a Frequent Oligogenic Involvement in Primary Ovarian Insufficiency Onset

**DOI:** 10.3389/fendo.2021.664645

**Published:** 2021-11-04

**Authors:** Raffaella Rossetti, Silvia Moleri, Fabiana Guizzardi, Davide Gentilini, Laura Libera, Anna Marozzi, Costanzo Moretti, Francesco Brancati, Marco Bonomi, Luca Persani

**Affiliations:** ^1^ Department of Endocrine and Metabolic Diseases and Lab of Endocrine and Metabolic Research, Istituto di Ricovero e Cura a Carattere Scientifico (IRCCS) Istituto Auxologico Italiano, Milan, Italy; ^2^ Molecular Biology Laboratory, IRCCS Istituto Auxologico Italiano, Milan, Italy; ^3^ Bioinformatics and Statistical Genomics Unit, IRCCS Istituto Auxologico Italiano, Milan, Italy; ^4^ Department of Medical Biotechnologies and Translational Medicine, University of Milan, Milan, Italy; ^5^ Department of Systems Medicine, Tor Vergata University, Rome, Italy; ^6^ Medical Genetics, Department of Life, Health and Environmental Sciences, University of L’Aquila, L’Aquila, Italy; ^7^ Human Functional Genomics, IRCCS San Raffaele Pisana, Rome, Italy

**Keywords:** primary ovarian insufficiency, primary amenorrhea, secondary amenorrhea, oligogenic disease, next-generation sequencing

## Abstract

Primary ovarian insufficiency (POI) is one of the major causes of female infertility associated with the premature loss of ovarian function in about 3.7% of women before the age of 40. This disorder is highly heterogeneous and can manifest with a wide range of clinical phenotypes, ranging from ovarian dysgenesis and primary amenorrhea to post-pubertal secondary amenorrhea, with elevated serum gonadotropins and hypoestrogenism. The ovarian defect still remains idiopathic in some cases; however, a strong genetic component has been demonstrated by the next-generation sequencing (NGS) approach of familiar and sporadic POI cases. As recent evidence suggested an oligogenic architecture for POI, we developed a target NGS panel with 295 genes including known candidates and novel genetic determinants potentially involved in POI pathogenesis. Sixty-four patients with early onset POI (range: 10–25 years) of our cohort have been screened with 90% of target coverage at 50×. Here, we report 48 analyzed patients with at least one genetic variant (75%) in the selected candidate genes. In particular, we found the following: 11/64 patients (17%) with two variants, 9/64 (14%) with three variants, 9/64 (14%) with four variants, 3/64 (5%) with five variants, and 2/64 (3%) with six variants. The most severe phenotypes were associated with either the major number of variations or a worse prediction in pathogenicity of variants. Bioinformatic gene ontology analysis identified the following major pathways likely affected by gene variants: 1) cell cycle, meiosis, and DNA repair; 2) extracellular matrix remodeling; 3) reproduction; 4) cell metabolism; 5) cell proliferation; 6) calcium homeostasis; 7) NOTCH signaling; 8) signal transduction; 9) WNT signaling; 10) cell death; and 11) ubiquitin modifications. Consistently, the identified pathways have been described in other studies dissecting the mechanisms of folliculogenesis in animal models of altered fertility. In conclusion, our results contribute to define POI as an oligogenic disease and suggest novel candidates to be investigated in patients with POI.

## 1 Introduction

Female factors account for one-third of all causes of infertility. Besides tubal disease and endometrial pathology, the dysregulation of any essential step involved in the ovulation of a competent oocyte may cause primary ovarian insufficiency (POI), a clinical syndrome defined by the premature loss of ovarian function. A recent meta-analysis of 31 epidemiological studies on the prevalence of POI in different countries between 1987 and 2018 reports an overall occurrence up to 3.7% in women younger than 40 years (1). At present, this disease is currently diagnosed when fertility is irreversibly affected. POI can manifest with a wide variety of clinical phenotypes, ranging from ovarian dysgenesis (OD) and primary amenorrhea (PA) to post-pubertal secondary amenorrhea (SA) for more than 4 months with raised gonadotrophins and low estradiol. The consequent long-standing estrogen deficiency exposes these women to an increased risk of complications such as cardiovascular diseases, reduced bone mineral density, and cognitive impairment (2). This disorder is highly heterogeneous in its etiology and several causes have been reported, mainly genetic, associated with chromosomal abnormalities (especially including X chromosome, such as in Turner syndrome), but also autoimmune, infectious, or iatrogenic. However, most causes of POI are still unknown, and the identification of novel causative genes is challenging. More recently, the advent of next-generation sequencing (NGS) technique and, especially, the whole exome screening (WES) of large POI families expanded the list of candidate genes to be screened in patients and consequently empowered the possibilities of a genetic diagnosis in idiopathic cases (3). Some WES studies demonstrated that pathogenic variants in meiotic chromosome pairing and synaptonemal complex (4–6) or alterations of other proteins of DNA recombination and repair (7, 8) could be responsible for POI onset by usually impairing meiotic progression and triggering oocyte death, as further evidenced by murine models (9). Other studies identified variants in the folliculogenesis players of all stages of ovarian follicle maturation, which involves the precise interaction of hundreds of genes: from the primordial follicle stock establishment of ovarian reserve (10) to the primordial to primary follicle activation (11, 12), throughout the follicular development in the gonadotropin-independent (13, 14) and gonadotropin-dependent stages (15, 16). Furthermore, alterations in genes contributing to extracellular matrix (ECM) remodeling by proteolytic activity on specific substrates within the ovarian context have been linked to the ECM turnover of abnormal somatic cells, thus leading to defects in follicular development (17). Since biological processes related to metabolism and immune system activation resulted to be enhanced in gene expression dynamics along with ovary development, including pathways associated with cell cycle, proliferation, apoptosis, ovulation, angiogenesis, and steroidogenesis (18), the disturbance of any of these pathways has been associated with reproductive diseases like POI (19–22). Moreover, the remarkable point that emerged from recent NGS studies is the occurrence of oligogenic defects (6, 23). From this perspective, various interacting genes might affect several mechanisms and pathways, and the synergistic and/or cumulative effect of several variants may contribute to POI phenotype. The NGS results in 64 patients with PA or early onset SA based on our panel of 295 POI candidate genes presented here aims to contribute to expand the list of potentially causative candidate genes and to support a genetically heterogeneous architecture of POI, resulting from defects in multiple complementary pathways.

## 2 Materials and Methods

### 2.1 Next-Generation Sequencing Panel Construction and Analysis

Genomic DNA (gDNA) was extracted from peripheral blood of enrolled patients. The Ampliseq Custom DNA panel (Illumina) was designed *ad hoc* to include the coding exons and flanking splice sites of 295 genes. The NGS panel, called *OVO-Array* (**Table S1**), has been obtained by joining data from: a) literature search of genes known to be previously associated with female infertility, with a role in ovary and in menopause onset (*n =* 159) (23); b) transcriptomic analysis of human granulosa cells treated with the oocyte-derived growth factor BMP15 (*n* of genes: 19; focus of another study with manuscript under review); and c) WES on 10 Italian women selected with the most severe phenotypes of PA and OD, six of them with familial inheritance (*n =* 117). The latter were identified among a total of 18,570 rare variants (missense, 86%; nonsense, 3.5%; indels, 5.6%). Briefly, pathway analysis (by Reactome v.74) of 1,916 genetic identifiers, found mutated at least once in WES, revealed an enrichment in the following biological processes with a key role in ovarian functionality (e.g., chromatin organization, cell cycle and meiosis, extracellular matrix organization, and cell–cell communication). Within these pathways, we further selected 117 genes potentially correlated with POI onset. The total coverage of the target genes by the designed amplicons was 100%. Library was prepared using enzymatic DNA fragmentation, with 50 ng of total gDNA and quantified with Quant-iT PicoGreen (Thermo Fisher Scientific). Nextera Rapid Capture Enrichment protocol (Illumina) was followed to tagment gDNA, amplify tagmented gDNA, hybridize probes, capture hybridized probes and for library capture and amplification. The library was then loaded onto the reagent cartridge (Illumina) and sequencing was performed on a NextSeq 500 (Illumina). Reads were then examined to identify single nucleotide alterations or small insertions/deletions, and a first *in silico* analysis (including base calling and demultiplexing) has been performed using MiSeq provided software (Real Time Analysis RTA v.1.18.54 and Casava v.1.8.2, Illumina). FastQ files for each sample, containing mate paired-end reads after demultiplexing and adapter removal, have been used as input for MiSeq pipeline. Briefly, FastQ files have been processed with MiSeq Reporter v2.0.26 using the Custom Amplicon workflow. This analytical method required FastQ files, a “Manifest file” containing information about the sequences of primer pairs, the expected sequence of the amplicons, and the coordinates of the reference genome (*Homo sapiens*, hg19, build 37.2) as input. Each read pair has been aligned using the MEM algorithm of the BWA software. Local Indel realignment and base recalibration step were performed using the software GATK. The realigned and recalibrated BAM file was used as input to GATK Unified Genotyper thus generating a VCFv4.2 file for each sample. Quality control of sequencing data was performed directly on FastQ files using the FastQC software. Reads were also filtered based on quality mapping and removed if their quality mapping was <20. Genetic variants showing a PHredScore lower than 20 were also filtered out.

### 2.2 POI Patients

We collected a cohort of patients to be analyzed through the *OVO-Array* panel, following IRCCS Istituto Auxologico Italiano Ethics Committee approval (BIOEFFECT, code 05M101_2014), composed of a total of 64 women with either primary (PA, *n =* 21) or early onset secondary amenorrhea (SA, *n =* 43), with onset before 25 years, all characterized by FSH values >40 IU/L and low estradiol. We excluded karyotype abnormalities, FMR1 premutations, and ovarian autoimmunity in all of them. Moreover, we selected additional 43 patients (PA, *n =* 18 and SA, *n =* 25) with the same inclusion criteria and early onset POI before 25 years of age. These patients have been analyzed between the years 2013 and 2020 for variants on a selected subset of only nine causative genes for POI, mainly involved in folliculogenesis and meiosis (*BMP15*, *FIGLA*, *FOXL2*, *FSHR*, *GDF9*, *NOBOX*, *NR5A1*, *SYCE1*, *STAG3*) for diagnostic purposes at IRCCS Istituto Auxologico Italiano. The results obtained with the different screening approaches (*OVO-Array* vs. diagnostic routine) have been then compared. Informed consent was obtained from all patients prior to blood sample collection and molecular studies.

### 2.3 *In Silico* Analysis of NGS Results and Variant Interpretation

The genetic variants resulting from the *OVO-Array* experiment and those derived from the diagnostic routine were annotated using Annovar software (24) and then classified as *rare* if resulting unknown or with a minor frequency allele (MAF) <0.01 in 1000 genomes, dbSNP, or EXAC databases. By checking for pathogenicity prediction the VarSome database until July 2021 (25), only those rare variants classified as likely pathogenic (LP), pathogenic (P), or variant of unknown significance (VUS), according to the American College of Medical Genetics (ACMG) classification guidelines (26), were considered for further analysis and confirmed by using Sanger sequencing. Gene ontology analysis of all the significatively altered genes was performed against DAVID Bioinformatics v6.8 (27) and Reactome Pathway Browser version 74 (28) on October 26, 2020. Both databases cross-reference other resources (e.g., NCBI, Ensembl, UniProt, KEGG, ChEBI, PubMed, and GO).

## 3 Results

### 3.1 NGS Identified Novel Variants in Putative Candidates for POI

We recruited 64 patients presenting PA or SA with early onset (from 10 to 25 years) and performed NGS analysis on 295 selected genes, including known candidates and novel potentially causative genes. The mean coverage depth of the target regions was greater than 90% at 50× for all patients. Variants with MAF >0.01 were filtered out. We considered the identified variants relevant only if they were *rare* (MAF <0.01) or never described; predicted as P, LP, or VUS; and already known to be associated with POI. We also considered nine variants predicted as likely benign, found in recurring genes with a higher frequency in our patients than in the general female population or previously associated to POI and functionally characterized.

**Figure 1:**
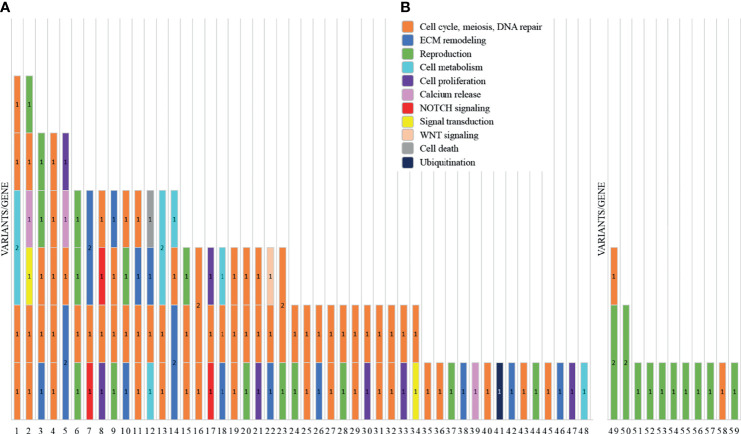
Graph representations of the number of variants identified by NGS analysis in two subgroups of primary ovarian insufficiency (POI) patients. For each patient, indicated are the ID number (*x*-axis) and the number of variants/genes found altered (1 = 1 variant/gene; 2 = 2 variants/gene). Each color represents a different pathway: cell cycle, meiosis, and DNA repair (orange); ECM remodeling (blue); reproduction (green); cell metabolism (cyan); cell proliferation (purple); calcium release (pink); NOTCH signaling (red); signal transduction (yellow); WNT signaling (light orange); cell death (gray); and ubiquitination (dark blue). **(A)** The histogram reports the variants found by screening 295 candidate genes included in the *OVO-Array* panel. **(B)** NGS results of the analysis for diagnostic purposes of nine known POI genes.

**Figure 2:**
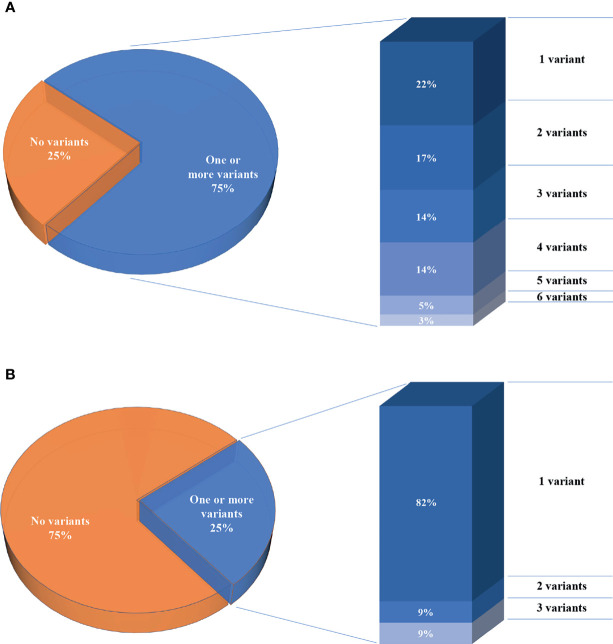
Percentages of the number of patients per number of variants. **(A)** Seventy-five percent of patients analyzed through the *OVO-Array* panel harbor one or more variants, left. Histogram representing the percentages of patients carrying variants, right. **(B)** Twenty-five percent of patients analyzed in only nine known POI genes.

We report a total of 114 rare variants in 78 different genes (**Figure 1A**, **Table S2**) in 64 patients. In 75% of patients analyzed by means of *OVO-Array*, we could find at least one alteration (48/64) possibly related to POI. Noteworthy, 2 or more variants have been identified in 34 of them; in particular, we found 11 patients (11/64, 17%) with two variants, 9 patients (9/64, 14%) with three variants, 9 patients (9/64, 14%) with four variants, 3 patients (3/64, 5%) with five variants, and 2 patients (2/64, 3%) with six variants (**Figure 2A**, **Table S3**). No significative alterations in the selected 295 genes were found in only 16 patients of the cohort.

Moreover, among the 43 patients screened for diagnostic purposes, we could identify at least one genetic variant in known POI genes in 11 of them, thus providing a genetic diagnosis in about 25% of POI patients through NGS. Only one patient harbored two different alterations and another one harbored three variants (**Figures 1B**, **2B**, **Tables S4**, **S5**). All the identified variants were confirmed by Sanger sequencing.

#### 3.1.1 Correlation Among the Number of Variants per Patient, Predicted Pathogenicity, and Phenotype

We summarized the prioritized variants harbored by each patient analyzed by *OVO-Array* NGS panel in **Supplementary Tables S2** and **S3**. The phenotype of each patient is also reported. Nomenclature validation was performed using Mutalyzer 2.0.33 (https://www.mutalyzer.nl/) according to HGVS nomenclature version 2.0.

**Tables 1**–**6** display all the genetic variations identified by *OVO-Array* by the number of variants per patient. Briefly, each table prioritizes patients depending on their phenotype severity and shows a detailed characterization (including origin, karyotype, age of menarche and POI onset, and familiarity). The frequency of each variant is then compared with those of the female general population available in the gnomAD ver.2.1.1 public dataset. The number of variants per patient is thus correlated with the VarSome predicted pathogenicity and phenotype of the patients. For each variation, VarSome criteria and its relative updated link are shown.

**Table 1:** Correlation among severity, phenotype and pathogenicity predictions in patients with 6 variants in potentially POI genes.

Patients ID	Origin	Karyotype	Phenotype	Menarche(yrs)	POI onset(yrs)	Familiarity	Variation (HGVS)	Zigosity	OVO-Array patients’ frequency (n = 64)	gnomAD ver. 2.1.1 female population frequency	VarSome prediction v.2.1.1	VarSome Criteria
1	Caucasian	46,XX	PA	16 (induced)	16	No	LARS2(NM_015340.4):c.1021T>G:(p.Cys341Gly)	Het	0.015625	Novel	VUS	PM2,PP2,BP4
							LARS2(NM_015340.4):c.1717T>C:(p.Phe573Leu)	Het	0.015625	Novel	VUS	PM2,PP2,PP3
							MLH3(NM_001040108.2):c.3466G>A:(p.Val1156Ile)	Het	0.015625	0.0000696	VUS	PP3,BP1
							POLE(NM_006231.4):c.4900C>T:(p.Arg1634Cys)	Het	0.015625	0.00000868	VUS	PM2,PP3,BP1
							ANAPC1(NM_022662.4):c.2279C>G:(p.Pro760Arg)	Het	0.015625	0.0000607	VUS	PM2,PP3,BP1
							ATR(NM_001184.4):c.2783A>G:(p.Gln928Arg)	Het	0.015625	Novel	VUS	PM2,BP1
2	Caucasian	46,XX	eSA	12	12 (6 month after menarche)	No	COL6A2(NM_001849.4):c.511G>A:(p.Gly171Arg)	Het	0.015625	0.00114	VUS	PM2,PP2,PP3
							ATM(NM_000051.4):c.1444A>C:(p.Lys482Gln)	Het	0.015625	0.000104	VUS	PM2,BP4
							RASAL2(NM_004841.5):c.3187G>A:(p.Glu1063Lys)	Het	0.015625	0.000338	VUS	PP3,BS1
							RYR3(NM_001036.6):c.14584C>T:(p.Arg4862Cys)	Het	0.015625	0.0000175	VUS	PM2,PP3,BP1
							TP73(NM_005427.4):c.1660T>C:(p.Tyr554His)	Het	0.015625	–	VUS	PM2,PP3
							CYP21A2(NM_000500.9):c.844G>T:(p.Val282Leu)	Hom	0.03125	–	P	PS1,PS3,PM2,PP2,PP5,BP4
							de novo microdeletion of 246kb in 1p36.33 (hg19, chr1:1.228.154-1.473.662 Mb) devoid of known POI genes	Het	0.015625	–	–	–

PA, primary amenorrhea; eSA, early secondary amenorrhea; Het, heterozygosity; Hom, homozygosity; VUS, variant of unknown significance; P, pathogenic.

**Table 2:** Correlation among severity, phenotype and pathogenicity predictions in patients with 5 variants in potentially POI genes.

Patients ID	Origin	Karyotype	Phenotype	Menarche(yrs)	POI onset(yrs)	Familiarity	Variation (HGVS)	Zigosity	OVO-Array patients’ frequency (n = 64)	gnomAD ver. 2.1.1 female population frequency	VarSome prediction v.2.1.1	VarSome Criteria
3	Caucasian	46,XX	OD	No	–	Yes	CYP21A2(NM_000500.9):c.1360C>T:(p.Pro454Ser)	Het	0.015625	–	P	PP5,PS3,PM2,PP2,PP3
							ATM(NM_000051.4):c.418G>C:(p.Asp140His)	Het	0.015625	Novel	VUS	PM2,PP3,BP6
							BLM(NM_000057.4):c.2333C>G:(p.Ser778Cys)	Het	0.015625	0.000156	VUS	PM1,PM2,PP3
							ADAMTS16(NM_139056.4):c.2459G>A:(p.Arg820Gln)	Het	0.015625	0.0000175	VUS	PP3,BS1
							FSHR(NM_000145.4):c.847C>T:(p.Arg283Trp)	Het	0.015625	0.000052	VUS	PM2,PP2
4	Caucasian	not available	PA	No	–	Yes	RYR3(NM_001036.6):c.393C>A:(p.Asp131Glu)	Het	0.015625	–	VUS	PP3,BP1
							NCOR2(NM_006312.6):c.3760C>T:(p.Arg1254Cys)	Het	0.015625	0.000123	VUS	PP3,BS1
							RAD52(NM_134424.4):c.761C>T:(p.Thr254Met)	Het	0.015625	0.000149	VUS	PP3
							MCM9(NM_017696.3):c.970G>T:(p.Val324Leu)	Het	0.015625	Novel	VUS	PM2,BP1
							RAD54L(NM_003579.4):c.604C>T:(p.Arg202Cys)	Het	0.015625	0.00324	LB	PM1,PP3,BS1,BP1,BP6
5	Caucasian	46,XX	SA	13	24	Yes	ERBB4(NM_005235.3):c.95C>T:(p.Thr32Met)	Het	0.015625	–	VUS	PM2,PP2,PP3
							ERBB4(NM_005235.3):c.3344T>A:(p.Val1115Glu)	Het	0.015625	Novel	VUS	PM2,PP2,BP4
							PKP1(NM_000299.3):c.883C>G:(p.Leu295Val)	Het	0.015625	0.00014	VUS	PM2,PP3,BP1
							RYR3(NM_001036.6):c.592A>G:(p.Met198Val)	Het	0.015625	–	VUS	PM2,BP1
							SAMD11(NM_152486.4):c.682_683insT: (p.Pro228LeufsTer227)	Het	0.09375	0.000183	LB	BS1

OD, ovarian dysgenesis; PA, primary amenorrhea; SA, secondary amenorrhea; Het, heterozygosity; P, pathogenic; VUS, variant of unknown significance; LB, likely benign.

**Table 3:** Correlation among severity, phenotype and pathogenicity predictions in patients with 4 variants in potentially POI genes.

Patients ID	Origin	Karyotype	Phenotype	Menarche(yrs)	POI onset(yrs)	Familiarity	Variation(HGVS)	Zigosity	OVO-Array patients’ frequency (n = 64)	gnomAD ver. 2.1.1 female population frequency	VarSome predictionv.2.1.1	VarSome Criteria
6	Caucasian	not available	OD	No	–	Yes	FIGLA(NM_001004311.3):c.364del:(p.Glu122LysfsTer45)	Het	0.015625	Novel	P	PVS1,PM2,PP5,PP3
							NOBOX(NM_001080413.3):c.1626del:(p.Phe543SerfsTer7)	Het	0.015625	Novel	P	PVS1,PM2,PP5
							NR5A1(NM_004959.5):c.1063G>A:(p.Val355Met)	Het	0.03125	0.000122	LP	PM1,PM2,PP5,PP2,PP3
							NCOR2(NM_006312.6):c.3755C>A:(p.Pro1252Gln)	Het	0.015625	Novel	VUS	PM2,PP3
7	Caucasian	not available	PA	No	–	No	VWF(NM_000552.5):c.4517C>T:(p.Ser1506Leu)	Het	0.046875	–	P	PP5,PM1,PM2,PP2,PP3
							VWF(NM_000552.5):c.4508T>C:(p.Leu1503Pro)	Het	0.03125	Novel	LP	PM1,PM2,PM5,PP2,PP3
							NOTCH3(NM_000435.3):c.2791A>G:(p.Ser931Gly)	Het	0.015625	Novel	LP	PM1,PM2,PP2,PP3
							TRRAP(NM_001244580.1):c.10171A>G:(p.Met3391Val)	Het	0.015625	Novel	VUS	PM2,PP2
8	Caucasian	not available	eSA	16	18	No	SAMD11(NM_152486.4):c.682_683insT: (p.Pro228LeufsTer227)	Het	0.09375	0.000183	LB	BS1
							ATG4C(NM_032852.4):c.607dup: (p.Trp203LeufsTer4)	Het	0.015625	0.000659	VUS	PP3,BS1
							NOTCH4(NM_004557.4):c.2945C>T:(p.Thr982Ile)	Het	0.015625	Novel	VUS	PM2,PP3,BP1
							RMI1(NM_024945.3):c.746C>T:(p.Ala249Val)	Het	0.015625	Novel	VUS	PM2,PP3
9	Caucasian	not available	eSA	14	14	No	NR5A1(NM_004959.5):c.502G>C:(p.Ala168Pro)	Het	0.015625	Novel	VUS	PM2,PP2,BP4
							SYNE1(NM_182961.4):c.16709A>G:(p.Gln5570Arg)	Het	0.015625	Novel	LP	PP3,PM2
							RAD50(NM_005732.4):c.2165dup:(p.Glu723GlyfsTer5)	Het	0.015625	0.000301	P	PVS1,PP5,PM2,PP3
							ADAMTS5(NM_007038.5):c.1729G>A:(p.Gly577Ser)	Het	0.03125	0.0151	VUS	PP3,BS1
10	Caucasian	46,XX	SA	13	25	–	ADAMTS4(NM_005099.6):c.803G>A:(p.Arg268Gln)	Het	0.015625	–	VUS	PM2,BP4
							DHCR24(NM_014762.4):c.1046C>G:(p.Pro349Arg)	Het	0.015625	0.0000087	VUS	PM2,PP2,PP3
							FSHR(NM_000145.4):c.491C>T:(p.Ser164Phe)	Het	0.015625	Novel	VUS	PM2,PP2,PP3
							RAD52(NM_134424.4):c.175G>A:(p.Gly59Arg)	Het	0.015625	0.000699	VUS	PP3
11	Caucasian	46,XX	SA	11	14	No	APC2(NM_005883.3):c.2887C>T:(p.Arg963Trp)	Het	0.015625	0.0000135	VUS	PM2,PP3,BP1
							ATG2A(NM_015104.3):c.4414G>C:(p.Gly1472Arg)	Het	0.015625	0.0000174	VUS	PM2,PP3,BP1
							COL6A1(NM_001848.3):c.350T>C:(p.Val117Ala)	Het	0.015625	0.000982	VUS	PM2,PP2,PP3
							KPNA2(NM_002266.4):c.445T>C:(p.Ser149Pro)	Het	0.015625	Novel	VUS	PM2.PP3
12	Caucasian	not available	SA	11	25	No	LARS2(NM_015340.4):c.2192A>T:(p.Tyr731Phe)	Het	0.015625	Novel	VUS	PM2,PP2,BP4
							RBBP8(NM_002894.3):c.2516G>A(p.Arg839Gln)	Het	0.03125	0.000415	VUS	PM2,PP3,BP1
							THBS2(NM_003247.5):c.1183G>A:(p.Val395Met)	Het	0.015625	0.0000528	VUS	PP3,BS1,BP1
							RIPK1(NM_003804.6):c.700G>A:(p.Glu234Lys)	Het	0.015625	0.000684	VUS	PM2,PP2,BP4,BP6
13	Caucasian	not available	SA	12	21	No	ATM(NM_000051.4):c.7375C>T:(p.Arg2459Cys)	Het	0.015625	0.0000433	VUS	PM1,PM2,PP3
							NCOR2(NM_006312.6):c.3709G>A:(p.Val1237Ile)	Het	0.015625	0.00000886	VUS	PP3
							POLG(NM_001126131.2):c.752C>T:(p.Thr251Ile)	Het	0.03125	0.00147	LP	PS3,PM2,PP2,PP5
							POLG(NM_001126131.2):c.1760C>T:(p.Pro587Leu)	Het	0.03125	0.0015	P	PP5,PS3,PM2,PP2,PP3
14	Caucasian	46,XX	SA	12	16	Yes	VWF(NM_000552.5):c.4517C>T:(p.Ser1506Leu)	Het	0.046875	Novel	P	PP5,PM1,PM2,PP2,PP3
							VWF(NM_000552.5):c.5641G>A:(p.Asp1881Asn)	Het	0.015625	Novel	VUS	PM2,PP2,PP3
							LGR4(NM_018490.5):c.2531A>G:(p.Asp844Gly)	Het	0.015625	0.0124	VUS	PP3,BS1,BP1,BP6
							HK3(NM_002115.3):c.2389G>A:(p.Glu797Lys)	Het	0.015625	0.000113	VUS	PP3,BS1,BP1

OD, ovarian dysgenesis; PA, primary amenorrhea; eSA, early secondary amenorrhea; SA, secondary amenorrhea; Het, heterozygosity; P, pathogenic; VUS, variant of unknown significance; LP, likely pathogenic; LB, likely benign.

**Table 4:** Correlation among severity, phenotype and pathogenicity predictions in patients with 3 variants in potentially POI genes.

Patients ID	Origin	Karyotype	Phenotype	Menarche(yrs)	POI onset(yrs)	Familiarity	Variation (HGVS)	Zigosity	OVO-Array patients’ frequency (n = 64)	gnomAD ver. 2.1.1 female population frequency	VarSome predictionv.2.1.1	VarSome Criteria
15	Caucasian	not available	PA	9	9	Yes	TEX15(NM_001350162.2):c.6511C>T:(p.Arg2171Ter)	Het	0.015625	0.0000175	p	PVS1,PM2,PP3
							TUBA8(NM_018943.3):c.967G>A:(p.Val323Met)	Het	0.015625	0.000243	VUS	PM2,PP3,BP1
							ID1(NM_002165.4):c.458_460del:(p.Leu153del)	Het	0.015625	0.00000886	VUS	PM2,PM4,BP4
16	Caucasian	not available	PA	–	–	No	ATR(NM_001184.4):c.4610T>A:(p.Leu1537Ter)	Het	0.015625	Novel	P	PVS1,PM2,PP3
							POLG(NM_001126131.2):c.1760C>T:(p.Pro587Leu)	Het	0.03125	0.0015	P	PP5,PS3,PM2,PP2,PP3
							POLG(NM_001126131.2):c.752C>T:(p.Thr251Ile)	Het	0.03125	0.00147	LP	PS3,PM2,PP2,PP5
17	Caucasian	46,XX	eSA	12	12	No	NOTCH2(NM_024408.4):c.2084C>A:(p.Ala695Glu)	Het	0.015625	Novel	VUS	PM2,PP3
							TP53(NM_001126114.2):c.475G>A:(p.Ala159Thr)	Het	0.015625	–	P	PM1,PM2,PM5,PP2,PP3
							SAMD11(NM_152486.4):c.682_683insT: (p.Pro228LeufsTer227)	Het	0.09375	0.000183	LB	BS1
18	Caucasian	46,XX	SA	13	19	No	ERBB3(NM_001982.4):c.2269dup:(p.Thr757AsnfsTer70)	Het	0.015625	Novel	P	PVS1,PM2,PP3
							POLG(NM_001126131.2):c.803G>C:(p.Gly268Ala)	Het	0.015625	0.00339	VUS	PP2,PP3,PP5,BS2
							LARS2(NM_015340.4):c.457A>C:(p.Asn153His)	Het	0.015625	–	LP	PP3,PM2,PP2,PP5
19	Caucasian	46,XX	SA	14	23	No	RAD52(NM_134424.4):c.388G>A:(p.Glu130Lys)	Het	0.015625	0.000138	VUS	PP3
							RBBP8(NM_002894.3):c.2516G>A:(p.Arg839Gln)	Het	0.03125	0.000415	VUS	PM2,PP3,BP1
							PLEC(NM_201380.4):c.5801G>A:(p.Arg1934His)	Het	0.015625	0.000128	VUS	PM2,PP3,BP1
20	Caucasian	46,XX	SA	13	22	No	ATG4C(NM_032852.4):c.774_777del:(p.Ile258MetfsTer13)	Het	0.015625	0.0000546	VUS	PP3,BS1
							ATM(NM_000051.4):c.4829G>C:(p.Arg1610Thr)	Het	0.015625	Novel	VUS	PM2,PP3
							PRIM1(NM_000946.3):c.911G>T:(p.Arg304Leu)	Het	0.015625	Novel	VUS	PM2,PP3
21	Caucasian	46,XX	SA	not available	19	No	SAMD11(NM_152486.4):c.682_683insT: (p.Pro228LeufsTer227)	Het	0.09375	0.000183	LB	BS1
							KMT2D(NM_003482.4):c.10876C>T:(p.Arg3626Trp)	Het	0.015625	–	VUS	PM2,PP3
							NBN(NM_002485.5):c.596C>G:(p.Pro199Arg)	Het	0.015625	–	VUS	PM2,PP3
22	Caucasian	46,XX	SA	not available	20	No	COL6A2(NM_001849.4):c.343C>T:(p.Arg115Trp)	Het	0.015625	0.00000873	VUS	PM2,PP2,PP3
							LRP5(NM_002335.4):c.4511C>T:(p.Pro1504Leu)	Het	0.015625	0.000389	VUS	PM2,PP2,PP3,BP6
							APC2(NM_005883.3):c.932C>T:(p.Ser311Leu)	Het	0.015625	–	VUS	PM2,PP3,BP1
23	Caucasian	46,XX	SA	14	16	not available	BMP15(NM_005448.2):c.202C>T:(p.Arg68Trp)	Het	0.015625	0.000708	B	PM1,PP3,PP5,BS1,BS2,BP1
							GDF9(NM_005260.5):c.1121C>T:(p.Pro374Leu)	Het	0.015625	0.0000346	VUS	PM2,PP3,BP6
							GDF9(NM_005260.5):c.278A>G:(p.Tyr93Cys)	Het	0.015625	Novel	VUS	PM1,PM2,PP3

PA, primary amenorrhea; eSA, early secondary amenorrhea; SA, secondary amenorrhea; Het, heterozygosity; P, pathogenic; VUS, variant of unknown significance; LP, likely pathogenic; LB, likely benign; B, benign.

**Table 5:** Correlation among severity, phenotype and pathogenicity predictions in patients with 2 variants in potentially POI genes.

Patients ID	Origin	Karyotype	Phenotype	Menarche(yrs)	POI onset(yrs)	Familiarity	Variation(HGVS)	Zigosity	OVO-Array patients’ frequency (n = 64)	gnomAD ver. 2.1.1 female population frequency	VarSome predictionv.2.1.1	VarSome Criteria
24	African	46,XX	OD	No	–	Yes	NOBOX(NM_001080413.3):c.1112A>C:(p.Lys371Thr)	Het	0.015625	–	B	BS1,BS2,BP1,BP4
							STAG3(NM_012447.4):c.1079G>A:(p.Arg360His)	Het	0.015625	–	LP	PM1,PM2,PP2,PP3
25	African	not available	PA	No	–	No	REC8(NM_005132):c.899G>T:(p.Arg300Leu)	Het	0.015625	0.00234	VUS	PPP3,BS1
							LHCGR(NM_000233):c.C568A:(p.Gln190Lys)	Het	0.015625	0.000563	VUS	PM2,PP2,BP6
26	Caucasian	46,XX	PA	No	–	Yes	AGRN(NM_198576.4):c.2860G>A:(p.Ala954Thr)	Het	0.015625	0.000026	VUS	PM2,PP3,BP1
							VLDLR(NM_003383.5):c.902G>A:(p.Arg301Gln)	Het	0.015625	0.00192	VUS	PM2,BP1
27	Caucasian	46,XX	PA	No	–	Yes	TP63(NM_003722.5):c.1927C>T:(p.Arg643Ter)	Het	0.015625	Novel	P	PVS1,PM2,PP3,PP5
							FANCA(NM_000135.4):c.1340C>T:(p.Ser447Leu)	Het	0.015625	0.000486	VUS	PM2,PP2,PP3
28	Caucasian	46,XX	eSA	12	13	No	DMRT3(NM_021240.4):c.897dup:(p.Ala300ArgfsTer4)	Het	0.015625	–	P	PVS1,PM2,PP3
							RELN(NM_005045.4):c.2015C>T:(p.Pro672Leu)	Het	0.015625	0.000139	LP	PM1,PM2,PP3,PP5,BP1
29	Caucasian	46,XX	eSA	13	13	No	HDAC5(NM_001015053.2):c.446A>G:(p.Glu149Gly)	Het	0.015625	–	VUS	PM2,PP3,BP1
							AKAP9(NM_005751.5):c.4351A>G:(p.Met1451Val)	Het	0.015625	–	VUS	PM2,PP3,BP1
30	Caucasian	46,XX	SA	9	22	Yes	SAMD11(NM_152486.4):c.682_683insT: (p.Pro228LeufsTer227)	Het	0.09375	0.000183	LB	BS1
							HK3(NM_002115.3):c.2077A>C:(p.Met693Leu)	Het	0.015625	0.000165	LB	PP3,BS1,BP1
31	Caucasian	46,XX	SA	12	22	not available	CCNB1IP1(NM_182852.3):c.454G>A:(p.Glu152Lys)	Het	0.015625	Novel	VUS	PM2,BP4
							MLH3(NM_001040108.2):c.3943G>A:(p.Glu1315Lys)	Het	0.015625	–	VUS	PM2,PP3,BP1
32	Caucasian	46,XX	SA	13	15	No	MSH4(NM_002440.4):c.1286A>T:(p.Glu429Val)	Het	0.015625	Novel	VUS	PM2,PP3
							CYP21A2(NM_000500.9):c.844G>T:(p.Val282Leu)	Het	0.03125	Novel	VUS	PP2,PP3
33	Caucasian	46,XX	SA	10	17	No	SAMD11(NM_152486.4):c.628C>T:(p.Arg210Cys)	Het	0.015625	0.000536	LB	PP3,BS1,BP1,BP6
							COL6A2(NM_001849.4):c.2575G>A:(p.Val859Met)	Het	0.015625	0.000241	VUS	PM2,PP2
34	Caucasian	46,XX	SA	10	25	No	GPR137C(NM_001099652.2):c.1211A>G:(p.Asp404Gly)	Het	0.015625	Novel	VUS	PM2,BP4
							RELN(NM_005045.4):c.3651C>G:(p.Ile1217Met)	Het	0.015625	0.00255	VUS	PM2,PP3,BP1

OD, ovarian dysgenesis; PA, primary amenorrhea; eSA, early secondary amenorrhea; SA, secondary amenorrhea; Het, heterozygosity; P, pathogenic; VUS, variant of unknown significance; LP, likely pathogenic; LB, likely benign; B, benign.

**Table 6:** Correlation among severity, phenotype, and pathogenicity predictions in patients with only 1 variant in potentially POI genes.

Patients ID	Origin	Karyotype	Phenotype	Menarche(yrs)	POI onset(yrs)	Familiarity	Variation (HGVS)	Zigosity	OVO-Array patients’ frequency (n = 64)	gnomAD ver. 2.1.1 female population frequency	VarSome predictionv.2.1.1	VarSome Criteria
35	Caucasian	not available	PA	No	–	No	RAD54L(NM_003579.4):c.2209C>A:(p.Gln737Lys)	Het	0.015625	Novel	VUS	PM2,PP3,BP1
36	Caucasian	not available	eSA	14	15	No	BRCA1(NM_007294.4):c.902A>G:(p.Lys301Arg)	Het	0.015625	Novel	VUS	PM2,PP3
37	Caucasian	46,XX	eSA	16	16	No	AR(NM_000044.6):c.2395C>G:(p.Gln799Glu)	Het	0.015625	0.00147	LP	PM1,PP2,PP3,PP5,BS2
38	Caucasian	46,XX	eSA	not available	Only menarche	No	COL6A2(NM_001849.4):c.2308G>C:(p.Glu770Gln)	Het	0.015625	Novel	VUS	PM2,PP2,PP3
39	Caucasian	not available	SA	12	25	Yes	RYR3(NM_001036.6):c.13709G>A:(p.Arg4570His)	Het	0.015625	0.00000877	VUS	PM2,PP3,BP1
40	Caucasian	not available	SA	12	18	No	CHEK2(NM_007194.4):c.1039G>A:(p.Asp347Asn)	Het	0.015625	0.0000259	LP	PM1,PM2,PM5,PP2,PP3,BP6
41	Caucasian	46,XX	SA	not available	15.5	Yes	USP35(NM_020798.4):c.1963dup:(p.Thr655AsnfsTer74)	Het	0.015625	0.0000267	VUS	PVS1,BS1
42	Caucasian	46,XX	SA	13	24	No	PKP1(NM_000299.3):c.2096A>T:(p.Lys699Met)	Het	0.015625	0.000156	VUS	PM2,PP3,BP1
43	Caucasian	not available	SA	12	18	No	KMT5A(NM_020382.7):c.287_289del:(p.Glu97del)	Het	0.015625	–	VUS	PM2,PM4,PP3
44	Caucasian	46,XX	SA	11	14	No	NR5A1(NM_004959.5):c.1063G>A:(p.Val355Met)	Het	0.03125	0.000122	LP	PM1,PM2,PP5,PP2,PP3
45	Caucasian	46,XX	SA	13	25	No	NCOA6(NM_014071.5):c.1250C>G:(p.Pro417Arg)	Het	0.015625	Novel	VUS	PM2,PP3,BP1
46	Caucasian	not available	SA	not available	18	Yes	ADAMTS5(NM_007038.5):c.1729G>A:(p.Gly577Ser)	Het	0.03125	0.0151	VUS	PP3,BS1
47	Caucasian	46,XX	SA	12	14	No	SAMD11(NM_152486.4):c.682_683insT: (p.Pro228LeufsTer227)	Het	0.09375	0.000183	LB	BS1
48	Caucasian	46,XX	SA	11	19	No	POLG(NM_001126131.2):c.3436C>T:(p.Arg1146Cys)	Het	0.015625	0.000182	VUS	PM2,PP2,PP3

PA, primary amenorrhea; eSA, early secondary amenorrhea; SA, secondary amenorrhea; Het, heterozygosity; VUS, variant of unknown significance; LP, likely pathogenic; LB, likely benign.

### 3.2 NGS Analysis Revealed Novel Rare Variants Affecting Pathways Involved in Ovarian Physiology

Taken together, our results uncovered 12 already known variants associated to POI: p.Arg300Leu in *REC8*; p.Arg68Trp in *BMP15*; p.Glu122Lysfs*45 in *FIGLA*; p.Pro103Ser, p.Thr121Ile, and p.Pro374Leu in *GDF9*; p.Phe543Serfs*7, p.Gly111Arg, and p.Lys371Thr in *NOBOX*; p.Val355Met in *NR5A1*; p.Arg643* in *TP63*; and p.Asn153His in *LARS2* (green colored genetic variants in **Tables S2**, **S4**). Moreover, we could identify 41 novel rare variants in genes already associated to POI etiology (red colored in **Tables S2**, **S4**) and 74 novel rare variants in additional genes participating in key steps of ovarian follicle development, which may be considered putative candidates involved in POI pathogenesis (**Table S2**). Interestingly, 74 variants have been identified in 27 recurring genes among our two POI populations, and some of these genes/variants have already been associated to POI: 9 genetic alterations and 10 genes (green and red colored in **Table 7**, respectively). Furthermore, we found 12 alterations in recurrent genes which affected more than one patient and resulted at higher frequency in our POI populations, with respect to the general female population reported in the gnomAD (ver. 2.1.1) database (**Table 7**): c.1729G>A (p.Gly577Ser) in *ADAMTS5*, c.202C>T (p.Arg68Trp) in *BMP15*, c.844G>T (p.Val282Leu) in *CYP21A2*, c.926G>C (p.Gly309Ala) in *FSHR*, c.1063G>A (p.Val355Met) in *NR5A1*, c.752C>T (p.Thr251Ile) and c.1760C>T (p.Pro587Leu) in *POLG*, c.2516G>A (p.Arg839Gln) in *RBBP8*, c.682_683insT (p.Pro228Leufs*227) in *SAMD11*, and c.4508T>C (p.Leu1503Pro) and c.4517C>T (p.Ser1506Leu) in *VWF*.

**Table 7:** Summary of the recurring variants in our POI cohorts.

Gene	Transcript	cDNA Variation	Protein Variation	PatientID	Pheno-type	POI group freq. (N=107)	gnomAD exomes (female) freq.	Varsome link	HGMD	Ref.
*ADAMTS5*	NM_007038	c.1729G>A	p.Gly577Ser	9	eSA	0.0186916	0.0151	varso.me/RpWE		
				46	SA					
*APC2*	NM_005883	c.2887C>T	p.Arg963Trp	11	SA	0.0093458	0.0000135	varso.me/TdbK		
		c.932C>T	p.Ser311Leu	22	SA	0.0093458	–	varso.me/Tdbc		
*ATG4C*	NM_032852	c.607dupT	p.Trp203Leufs*4	8	eSA	0.0093458	0.000659	varso.me/T0VM		
		c.774_777del	p.Ile258Metfs*13	20	SA	0.0093458	0.0000546	varso.me/T0Vd		
*ATM*	NM_000051	c.418G>C	p.Asp140His	3	46,XX OD	0.0093458	–	varso.me/LMjL		(29)
		c.1444A>C	p.Lys482Gln	2	eSA	0.0093458	0.000104	varso.me/FFvg		
		c.4829G>C	p.Arg1610Thr	20	SA	0.0093458	not available	varso.me/T0Bg		
		c.7375C>T	p.Arg2459Cys	13	SA	0.0093458	0.0000433	varso.me/SIfh		
*ATR*	NM_001184	c.4610T>A	p.Leu1537*	16	PA	0.0093458	not available	varso.me/TZxD		(30)
		c.2783A>G	p.Gln928Arg	1	PA	0.0093458	not available	varso.me/TZxU		
*BMP15*	NM_005448	c.202C>T	p.Arg68Trp	23	SA	0.0186916	0.000708	varso.me/Ne2w		(31)
				54	PA					
		c.406G>C	p.Val136Leu	55	PA	0.0093458	0.00000866	varso.me/TZn5		
*COL6A2*	NM_001849	c.343C>T	p.Arg115Trp	22	SA	0.0093458	0.00000873	varso.me/N8DA		
		c.511G>A	p.Gly171Arg	2	eSA	0.0093458	0.00114	varso.me/Orla		
		c.2308G>C	p.Glu770Gln	38	eSA	0.0093458	not available	varso.me/TZnl		
		c.2575G>A	p.Val859Met	33	SA	0.0093458	0.000241	varso.me/ReIP		
*CYP21A2*	NM_000500	c.844G>T	p.Val282Leu	2	eSA	0.0186916	not available	varso.me/N9iB	CM880022	
				32	SA					
		c.1360C>T	p.Pro454Ser	3	46,XX OD	0.0093458	not available	varso.me/Jl3r	CM920233	
*ERBB4*	NM_005235	c.3344T>A	p.Val1115Glu	5	SA	0.0093458	not available	varso.me/T1xM		(32)
		c.95C>T	p.Thr32Met			0.0093458	-	varso.me/T1x2		
*FSHR*	NM_000145	c.491C>T	p.Ser164Phe	10	SA	0.0093458	not available	varso.me/T1wg		(33)
		c.847C>T	p.Arg283Trp	3	46,XX OD	0.0093458	0.000052	varso.me/SWBI		
		c.926G>C	p.Gly309Ala	49	SA	0.0186916	not available	varso.me/S1eC		
				56	PA					
		c.1964G>C	p.Arg655Thr	49	SA	0.0093458	not available	varso.me/S1eT		
		c.909-917del	p.Tyr303*	50	SA	0.0093458	–	varso.me/TZcz		
		c.1118G>A	p.Ser373Asn			0.0093458	not available	varso.me/T1ug		
*GDF9*	NM_005260	c.362C>T	p.Thr121Ile	52	PA	0.0093458	0.000277	varso.me/TZ8s		(6, 34)
		c.566C>T	p.Thr189Ile	53	PA	0.0093458	not available	varso.me/TZ8M		
		c.307C>T	p.Pro103Ser	57	PA	0.0093458	0.0028	varso.me/TZ99		
		c.278A>G	p.Tyr93Cys	23	SA	0.0093458	not available	varso.me/Tjdt		
		c.1121C>T	p.Pro374Leu			0.0093458	0.0000346	varso.me/Tjdi	CM066831	
*HK3*	NM_002115	c.2077A>C	p.Met693Leu	30	SA	0.0093458	0.000165	varso.me/TjkN		(23)
		c.2389G>A	p.Glu797Lys	14	SA	0.0093458	0.000113	varso.me/TjkG		
*LARS2*	NM_015340	c.1021T>G	p.Cys341Gly	1	PA	0.0093458	not available	varso.me/TdCz		(35)
		c.1717T>C	p.Phe573Leu			0.0093458	not available	varso.me/TdEN		
		c.2192A>T	p.Tyr731Phe	12	SA	0.0093458	not available	varso.me/TdGx		
		c.457A>C	p.Asn153His	18	SA	0.0093458	not available	varso.me/R5je		
*MLH3*	NM_001040108	c.3466G>A	p.Val1156Ile	1	PA	0.0093458	0.0000696	varso.me/SR8H	CM1410381	
		c.3943G>A	p.Glu1315Lys	31	SA	0.0093458	–	varso.me/TjgS		
*NCOR2*	NM_006312	c.3755C>A	p.Pro1252Gln	6	46,XX OD	0.0093458	not available	varso.me/T2Av		
		c.3709G>A	p.Val1237Ile	13	SA	0.0093458	0.00000886	varso.me/T2BI		
		c.3760C>T	p.Arg1254Cys	4	PA	0.0093458	0.000123	varso.me/T2BR		
*NOBOX*	NM_001080413	c.331G>A	p.Gly111Arg	59	SA	0.0093458	0.0000131	varso.me/TZr6	CM152396	(36–38)
		c.1626delC	p.Phe543Serfs*7	6	46,XX OD	0.0093458	–	varso.me/T1kg		
		c.1112A>C	p.Lys371Thr	24	46,XX OD	0.0093458	0.000238	varso.me/SWXS		
*NR5A1*	NM_004959	c.1063G>A	p.Val355Met	6	46,XX OD	0.0186916	0.000122	varso.me/TdLE	CM076367	(36)
				44	SA					
		c.502G>C	p.Ala168Pro	9	eSA	0.0093458	-	varso.me/TdIj		
*PKP1*	NM_000299	c.883C>G	p.Leu295Val	5	SA	0.0093458	0.00014	varso.me/T2AC		
		c.2096A>T	p.Lys699Met	42	SA	0.0093458	0.000156	varso.me/T2Ao		
*POLG*	NM_001126131	c.752C>T	p.Thr251Ile	16	PA	0.0186916	0.00147	varso.me/FCE6	CM021660	(40)
				13	SA					
		c.803G>C	p.Gly268Ala	18	SA	0.0092592	0.00339	varso.me/IbzD	CM033442	
		c.1760C>T	p.Pro587Leu	16	PA	0.0186916	0.0015	varso.me/Ez4d	CM03133	
				13	SA					
		c.3436C>T	p.Arg1146Cys	48	SA	0.0093458	0.000182	varso.me/N8U8	CM060433	
*RAD52*	NM_134424	c.175G>A	p.Gly59Arg	10	SA	0.0093458	0.000699	varso.me/Td7L		
		c.761C>T	p.Thr254Met	4	PA	0.0093458	0.000149	varso.me/Td7a		
		c.388G>A	p.Glu130Lys	19	SA	0.0093458	0.000138	varso.me/Td7m		
*RAD54L*	NM_003579	c.604C>T	p.Arg202Cys	4	PA	0.0093458	0.00324	varso.me/FIC4		
		c.2209C>A	p.Gln737Lys	35	PA	0.0093458	not available	varso.me/TZvx		
*RBBP8*	NM_002894	c.2516G>A	p.Arg839Gln	12	SA	0.0186916	0.000415	varso.me/Fdvl		
				19	SA					
*RELN*	NM_005045	c.2015C>T	p.Pro672Leu	28	eSA	0.0093458	0.000139	varso.me/GFVk		
		c.3651C>G	p.Ile1217Met	34	SA	0.0093458	0.00255	varso.me/TCd9		
*RYR3*	NM_001036	c.393C>A	p.Asp131Glu	4	PA	0.0093458	–	varso.me/T1hi		
		c.592A>G	p.Met198Val	5	SA	0.0093458	–	varso.me/T1gq		
		c.14584C>T	p.Arg4862Cys	2	eSA	0.0093458	0.0000175	varso.me/T1fv		
		c.13709 G>A	p.Arg4570His	39	SA	0.0093458	0.00000877	varso.me/T1hS		
*SAMD11*	NM_152486	c.628C>T	p.Arg210Cys	33	SA	0.0093458	0.000536	varso.me/N60l		
		c.682_683insT	p.Pro228Leufs*227	8	eSA	0.0560748	0.000183	varso.me/N7qi		
				5	SA					
				17	eSA					
				47	SA					
				21	SA					
				30	SA					
*STAG3*	NM_012447	c.1079G>A	p.Arg360His	24	46,XX OD	0.0093458	–	varso.me/SWXv		(41)
		c.3433G>A	p.Glu1145Lys	49	SA	0.0093458	0.0000346	varso.me/S1e5		
		c.1678-10_2228del	p.?	58	PA	0.0093458	not available	varso.me/TZuL		
*VWF*	NM_000552	c.4508T>C	p.Leu1503Pro	7	PA	0.0093458	not available	varso.me/TYo1	CM095108	
		c.4517C>T	p.Ser1506Leu	7	PA	0.0186916	-	varso.me/TYnR	CM930731	
				14	SA					
		c.5641G>A	p.Asp1881Asn	14	SA	0.0093458	not available	varso.me/T1dI		

PA, Primary Amenorrhea; SA, Secondary Amenorrhea; eSA, early Secondary Amenorrhea; 46,XX OD, Ovarian Dysgenesis with normal karyotype (46,XX). Gene variations are shown, together with the patients’ phenotype. Genetic alterations genes and variants already associated to POI are shown in green and red characters, respectively. The frequency in our POI women (n= 107) and in the female population are given, together with the HGMD, if available.

### 3.3 Gene Ontology Analysis

**Figure 3:**
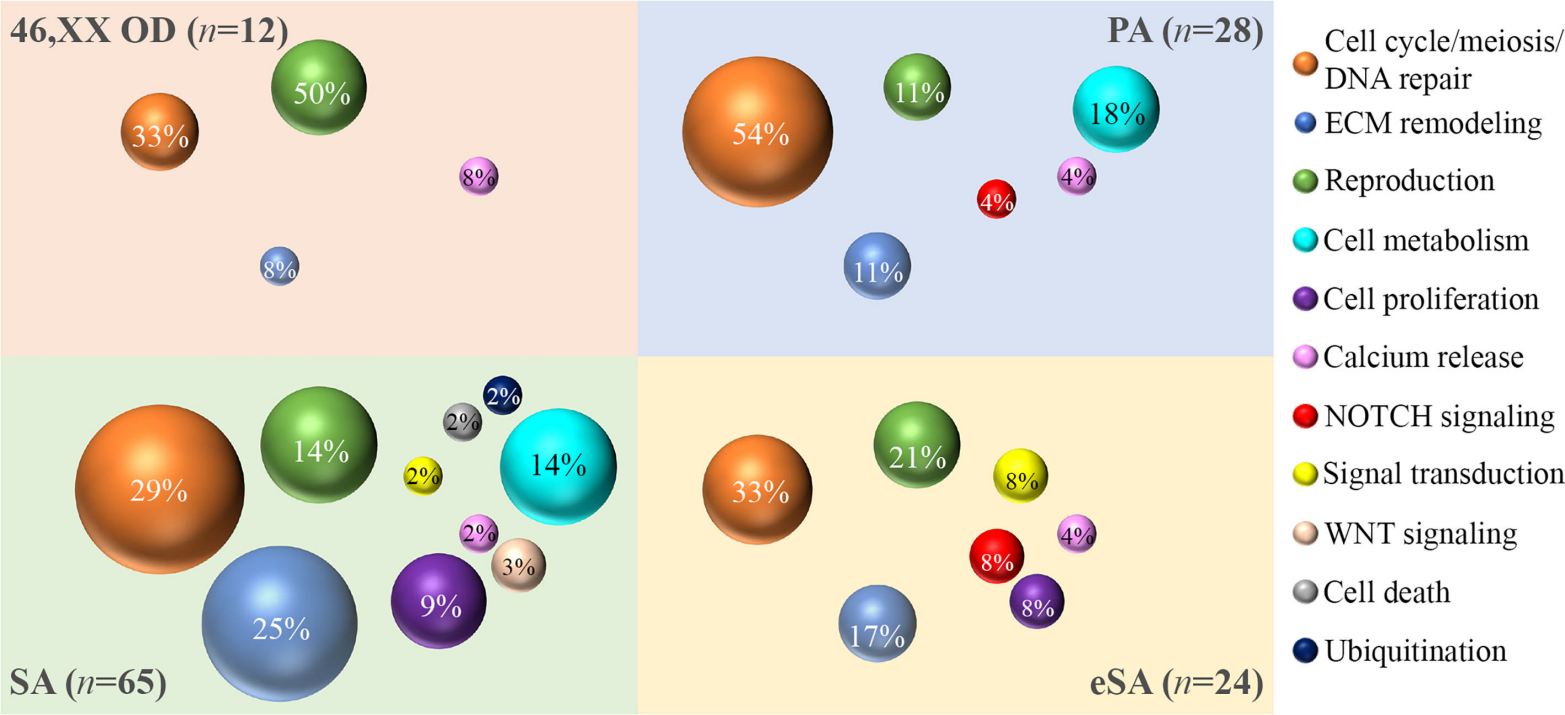
Bubble chart of the pathways identified by the gene ontology analysis. The sample numerosity (*n*) is reported for each phenotype (46,XX OD, light red; PA, light blue; eSA, light yellow; and SA <25 years, light green). Pathways are represented by colored bubbles (refer to the legend on the right). The size of the bubbles depends on the number of variants identified in each pathway for each phenotype. The percentages within the bubbles indicate the ratio between the number of variants per pathway associated to a phenotype and the total of variants associated to that phenotype. 46,XX OD = ovarian dysgenesis with normal karyotype (46,XX); PA = primary amenorrhea with functional ovarian defect, eSA = secondary amenorrhea onset few months after menarche; SA = secondary amenorrhea <25 years of age.

The gene ontology analysis was carried out by interrogating the Reactome pathway database and DAVID gene functional annotation tool (refer to **Tables S6**, **S7** for the statistical evaluation of the enrichment analysis). Considering both bioinformatic tools, we identified five main pathways likely affected by multiple genes found altered in our *OVO-Array* cohort and with functions in ovarian development and physiological maturation of oocytes (**Figure 1A**). Most altered genes, 34 in total, participated in meiosis, cell cycle, and DNA repair processes, whereas 15 genes were involved in reproductive pathways (folliculogenesis, oocyte maturation, and follicular development). Both groups resulted to be affected in all manifestations of POI, from 46,XX OD to SA, but especially in the most severe phenotypes. Twelve genes with variations were identified in the ECM remodeling pathway, which influences relevant processes during follicle development, such as cell morphology, communication, proliferation, survival, and steroidogenesis, and appeared to be mostly affected with later POI onset (SA <25 years), but also in few exceptional 46,XX OD or PA cases. Fourteen altered genes were involved in the control of specific aspects of cell metabolism with roles in ovarian function, in both PA and SA patients. A rare frameshift alteration of *SAMD11*, which was demonstrated as a promoter of cell proliferation, has been found in several patients mostly affected with SA. Four different variants of *RYR3*, coding for a calcium channel with a role in the homeostasis of calcium, have been identified among all phenotypes. Furthermore, another identified pathway, fundamental in granulosa cell differentiation and proliferation, is the NOTCH signaling, represented by *NOTCH2*, *NOTCH3*, and *NOTCH4* genes found to be altered in both PA and early SA cases. Finally, we also identified alterations in genes belonging to WNT signaling, cell death, and post-translational modifications mainly affecting SA patients (**Table S2**, **Figure 3**).

Most patients showed at least two pathways affected by variable genetic variations and a few showed several variations in genes from only one pathway (e.g., patient 27, presenting with PA and one variant in *TP63* and *FANCA* genes, both related to meiosis, cell cycle, and DNA repair processes). Among SA, we also found three patients with the involvement of several variations in a singular pathway: patient 19 with three variants and patient 31 with two variants affecting genes related to meiosis, cell cycle, and DNA repair processes and patient 23 with a variant in *BMP15* and a compound heterozygosity in *GDF9*, both key actors of folliculogenesis (**Table S3**).

## 4 Discussion

In this study, we used the *OVO-Array* panel, a targeted NGS approach, to investigate the genetic cause of POI in a cohort of 64 patients with early onset of the disorder. The sequencing was focused on 295 candidate genes selected from literature and our previous data. Through this approach, we could identify 9 already known variants (6, 31, 34, 36–38, 42, 43) and 32 novel rare variants in genes already associated to POI etiology. Moreover, 34 novel rare variants have been found in additional genes participating in pathways important for ovarian physiology.

### 4.1 The Oligogenic Nature of POI Is Suggested by Multilocus Analysis

Our data are consistent with several lines of evidence recently emerging pointing to an oligogenic architecture for POI, according to which the presence of multiple genetic variants may partially explain the heterogenicity of phenotypes observed in POI patients. Indeed, we observed at least two variants in 35 patients, 5% of the cohort screened with our *OVO-Array* panel. Noteworthy, two of these patients harbored six variants. On the contrary, the screening of nine POI genes for diagnostic routine permitted to identify only 2 out of the 11 patients with alterations (18%) carrying either two or three variants.

We observed that almost all patients screened by the *OVO-Array*, who carried the major number of variants, presented with a severe phenotype (PA or early SA onset just after menarche), whereas the majority of women carrying only one or two alterations (15 out of 25) were affected by SA. However, we also found that some patients harboring fewer pathogenic or likely pathogenic variants displayed a severe phenotype. These findings suggest that POI could emerge either by the disruption of a single fundamental genetic function or as a result of multilocus variations in genes interacting within different pathways, as proposed by other authors for oligogenic diseases (44). On this line, patient 6 of this cohort was previously reported for the potential synergic detrimental effect of a complex pattern of multiple inherited genetic variants in *FIGLA*, *NOBOX*, and *NR5A1* (36). In the present study, we included this patient into the *OVO-Array* analysis, and by sequencing additional genes, we could identify another alteration (c.3755C>A, p.Pro1252Gln) in *NCOR2*. This gene encodes for a nuclear receptor corepressor that, together with its paralog NCOR1, mediates the retinoic acid-dependent repression of Fgf8 in mouse during organogenesis and, if this complex is mutated, exhibits increased Fgf8 expression, similar to retinoic acid deficiency (45). On one hand, retinoic acid is critical for the entrance in meiosis of ovarian germ cells (46); on the other hand, FGF8 cooperates with BMP15 to promote glycolysis in cumulus cells (47). Therefore, we could hypothesize that an alteration affecting these fundamental pathways might combine with the other three already described variants and play a synergic effect leading to the severe phenotype of the patient. This evidence supports multilocus analysis as a fundamental tool to explain the full phenotypic spectrum of women with POI.

Since NGS has demonstrated to be a powerful tool for the identification of new molecular players and pathways in POI onset, the information derived from the analysis of large NGS panels, such as the *OVO-Array*, might increase the diagnostic power up to 75% of POI cases, in contrast to the current 25% of positive diagnosis obtained by screening few POI genes. The actual limit of this approach is the necessity of validating new putative candidates by further functional testing and sequencing in larger cohorts. Nonetheless, in this study, we could observe both the presence of the same variant in more than one patient at an increased frequency in women with POI with respect to the general female population and different alterations affecting the same genes in several patients. Although the fertility history of the controls reported in public datasets is unknown, thus introducing a potential bias in the analysis, our findings support the relevance of these candidates in POI pathogenesis and deserve further investigations.

### 4.2 Large-Scale Genetic Analysis Identified Novel Variants in Genes Participating in Pathways Relevant for Ovarian Function

A first hint pointing at the possible involvement of the newly identified candidates derives from gene ontology, which highlighted pathways fundamental for the reproductive success in mammals.

Meiotic recombination is a complex process, which requires the combination of a large plethora of factors (48). Furthermore, during the arrest at meiotic prophase I, primordial follicle oocytes are more vulnerable to DNA double stand breaks emanating from endogenous and exogenous sources (49); thus, pathogenic variations in one or more of their encoding genes might explain the onset of POI, as previously proposed (50). Noteworthy, we found 34 altered genes participating in meiosis, cell cycle, and DNA repair as possible candidates for POI. This pathway resulted to be mainly affected in patients with the most severe phenotypes, and in several cases, we found variants in at least two genes encoding DNA repair and meiotic factors. Moreover, this was the only pathway affected by genetic variants in some patients. Our data indicate that, in line with its oligogenic nature, POI might also result from alterations in more loci belonging to the same pathway.

The next major pathway in which we have found several altered genes was folliculogenesis, encompassing all stages of ovarian follicle development. In one case of SA (patient 23), we found variations in two major factors of folliculogenesis: a variant of *BMP15* (p.Arg68Trp) with deleterious functional effects (31), together with a compound heterozygosity in *GDF9* [p.Tyr93Cys and the previously reported p.Pro374Leu (34)], supporting the possibility that the disruption of a unique pathway at variable levels can also cause the disorder. Besides the known actors (i.e., *FIGLA*, *NOBOX*, *BMP15*, *GDF9*, *FSHR*, *LHCGR*, *NR5A1*, *AR*), we found variants in two genes involved in autophagosome assembly (*ATG2A* and *ATG4C*). Their role may deserve further attention because autophagy is an important mechanism in mammalian ovarian development, regulating follicular atresia (51). We also identified genes causing disorders of sex development or with roles in spermatogenesis. Indeed, *CYP21A2* alterations are responsible for congenital adrenal hyperplasia, caused by diminished aldosterone and cortisol production that result in ambiguous genitalia in female affected (52). *DMRT3* encodes for a transcription factor with evolutionary conserved roles in sex development, whose haploinsufficiency leads to 46,XY male to female sex reversal (53). *TUBA8* encodes an isoform of α-tubulin highly expressed in ovarian follicle (www.proteinatlas.org). This gene has an ortholog in mouse which is expressed in the brain and testis, with a role in spermatid development (54), and can cause asthenozoospermia in men (55). *ID1* has been described as potential AMH downstream target genes (56, 57), and since it is involved in the regulation of follicular growth, further characterization of its molecular function in this pathway would be needed. *KMT2D* alterations are the main cause of Kabuki syndrome, a congenital intellectual disability with genitourinary anomalies among other additional features. Interestingly, a reduced number of dominant families was reported in a Kabuki cohort (58), thus supporting a possible role of *KMT2D* variations in female fertility.

We also identified alterations in genes involved in ECM remodeling in the *OVO-Array* POI cohort, such as *ADAMTS4*, *ADAMTS5*, *ADAMTS16*, *AGRN*, *COL6A1*, *COL6A2*, *ERBB3*, *ERBB4*, *PKP1*, *RELN*, *THBS2*, and *VWF*. Given the ECM contribution to granulosa cell survival and proliferation, the ECM is required for maintaining the follicular cell morphology in all phases of ovarian follicle function (59). This ECM role is supposed to be mediated, at least in part, by the distinct ADAMTS subtypes and collagens. The ECM regulates cell aggregation and intracellular communication between the oocyte, granulosa, and theca cells within the follicle. The communication of small metabolites, ions, and second messengers between each cell type is made possible by a network of gap junctions, such as desmosomes containing among others the accessory plaque protein plakophilin 1. The ECM proteins are also necessary to support granulosa cell survival and steroidogenesis; for example, reelin contributes to follicular stability. Factors within the ECM are important in the control of follicular development and atresia. Included in this group of ECM proteins are thrombospondins, which are thought to contribute to the regulation of angiogenesis, and von Willebrand factor (59).

Several elements of Notch signaling appear to be involved in the POI pathogenesis in the *OVO-Array* cohort (*NOTCH2*, *NOTCH3*, *NOTCH4*). The Notch pathway is a contact-dependent signaling system active in the mammalian developing ovary which has multiple functions in follicle assembly, maturation, development, and meiotic entry (60). Notch proteins (NOTCH1, NOTCH2, NOTCH3, and NOTCH4) function as transmembrane receptors for one of the membrane-bound ligands (JAG1, JAG2, DLL1, DLL3, and DLL4) and their binding causes a conformational change of the Notch protein starting a series of sequential proteolytic cleavages at the receptor juxtamembrane region allowing the release of the Notch intracellular domain that is eventually free to translocate to the nucleus. Within the nucleus, it activates the transcription of Notch target genes. Receptors, ligands, modulators, and activated genes belonging to this system are expressed and finely regulated during folliculogenesis. Although Notch1 and Notch4 expression is limited to the ovarian vasculature, Notch2 and Notch3 are expressed in the granulosa cells of developing follicles and mediate follicle assembly and growth in mammals. Consistent with its role within the mammalian ovarian follicle, genetic alterations in *NOTCH2* were previously identified *via* whole-exome sequencing and functional evidence demonstrated the correlation of *NOTCH2* missense variations in POI (23, 61).

Several rare variants have been identified in genes regulating different aspects of follicular cell metabolism. Hexokinase 3 (HK3) converts glucose to glucose-6-phosphate in the first step of glucose metabolism, exerting a protective effect against oxidative stress (62), and it was associated with age at natural menopause (63). A recent transcriptomic study in primates demonstrated that genes most regulated in aged oocytes and granulosa cells are related to antioxidant defenses (64), further supporting the importance of cellular damage in infertility. *POLG* and *LARS2* play roles in mitochondrial DNA replication, gene expression, and protein synthesis and degradation (65). Mutations in *POLG* can cause among others the neurological conditions Alpers syndrome and progressive external ophthalmoplegia (PEO), and the latter can associate with POI (35). Four *POLG* variants emerged by our analysis: a PA (patient 23, see 3.1.1 section) and a SA patient with c. c.752C>T (p.Thr251Ile) and c.1760C>T (p.Pro587Leu) in compound heterozygosity; c.803G>C (p.Gly268Ala) and c.3436C>T (p.Arg1146Cys) in two patients with SA. Unfortunately, no further neurological information was available for these patients. Mutations in *LARS2*, encoding mitochondrial leucyl-tRNA synthetase, lead to POI and hearing loss in Perrault syndrome (66). We identified the c.457A>C (p.Asn153His) variant (ClinVar VCV000191173.2) of *LARS2* in a patient with SA, who was not previously associated with Perrault syndrome, and other three missense variants in two SA patients. Unfortunately, we have no further clinical information also in these patients. The cholesterol synthetase DHCR24 belongs to the pathway of steroid biosynthesis which regulates many physiological processes (i.e., stress response, ovarian cycle, and endocrine system) (67), and this is the first report of a variant in *DHCR24* in a patient with POI. The *VLDLR* gene in humans is relevant for steroidogenesis as its expression in granulosa cells of pre-ovulatory follicles keeps a relevant role in lipoprotein endocytosis during follicular growth (19). Evidence in hens correlates the VLDLR function with fertility (68) and its haploinsufficiency in women has been associated to impaired folliculogenesis (69). Here, we confirm previous findings by describing a novel missense variant in a patient with PA.

WNT signaling has essential functions in ovary differentiation, follicle development, and hormone synthesis (70). In this study, we identified missense variants in *LGR4*, one of the receptors for R-spondins, which augment the WNT signaling pathway (71), and *LRP5*, a co-receptor for the canonical Wnt–β-catenin signaling with a known role in the regulation of bone mineral density (72), but we could not find any further evidence in the ovary.

Cell proliferation and death pathways were represented by the identification in our cohort of variants in *SAMD11* and *RIPK1*, respectively. *SAMD11* has never been associated with ovarian phenotypes before. This gene encodes for a transcriptional modulator relatively uncharacterized but phylogenetically conserved from zebrafish to humans, widely expressed in various tissues (73), where it might promote cell proliferation. Conversely, *RIPK1* can mediate apoptosis during embryonic development (74). This is the first report of variants in these genes associated with an ovarian phenotype, and their role in the ovary should be investigated in further studies. Another gene involved in mitosis control that we found altered in POI was *USP35*, encoding a deubiquitinating enzyme which regulates the stability and function of Aurora B kinase and whose depletion results in inhibited metaphase chromosome alignment (75).

In mammalian oocytes, calcium is one of the major signal molecules involved in meiotic cell cycle resumption, arrest, and apoptosis. *RYR3* encodes a calcium channel that mediates the intracellular release of Ca^2+^, and the ryanodine receptors (RyR) family has been identified in mammalian oocytes (76). Here, we reported several *RYR3* variants with predicted pathogenic effect, which might dysregulate the homeostasis of calcium within oocytes, thus supporting a role for this gene in the disorder.

## 5 Conclusions

We propose novel candidate gene-disease variants likely causative or conferring susceptibility to POI onset. Our findings, by supporting the oligogenic nature of POI, suggest that in the presence of the most severe forms of ovarian insufficiency, it is necessary to screen multiple candidates or to perform WES analysis, since more alterations in different genes may synergize for the determination of the phenotype. Moreover, we show that multilocus analysis could increase the diagnostic power and the accuracy of POI diagnosis, thus ameliorating genetic counseling in patients. The systematic application of the *OVO-Array* multilocus analysis shall improve the management of POI including a personalized approach to the fertility defect and to the associated extra-ovarian abnormalities that can frequently anticipate or follow the POI onset.

## Data Availability Statement

The original contributions presented in the study are included in the article/**Supplementary Material** and online at https://doi.org/10.5281/zenodo.4543275 (Digital Object Identifier 10.5281/zenodo.4543275). Further inquiries can be directed to the corresponding authors.

## Ethics Statement

The studies involving human participants were reviewed and approved by IRCCS Istituto Auxologico Italiano Ethics Committee. Written informed consent to participate in this study was provided by the patients or, if minors, by the legal guardian/next of kin of the participants.

## Author Contributions

RR was responsible for the conception and design, *OVO-Array* experiment, and interpretation of data and drafted the manuscript. SM was responsible for the sequencing analysis, carried out the *in silico* predictions, and helped in drafting the manuscript. FG was responsible for NGS for diagnostic screening. DG was responsible for the bioinformatic NGS analysis. LL contributed to the *OVO-Array* experiment. AM, CM, FB, and MB were responsible for the enrollment of patients and critical revision of the manuscript. LP designed and supervised the research and revised the manuscript critically for important intellectual content. All authors contributed to the article and approved the submitted version.

## Funding

This research was funded by the Italian Ministry of Health with the grant “BIOEFFECT” (GR‐2011‐02351636 Giovani Ricercatori to RR). The funders had no role in the design of the study, data collection and analysis, decision to publish, or preparation of the manuscript.

## Conflict of Interest

The authors declare that the research was conducted in the absence of any commercial or financial relationships that could be construed as a potential conflict of interest.

## Publisher’s Note

All claims expressed in this article are solely those of the authors and do not necessarily represent those of their affiliated organizations, or those of the publisher, the editors and the reviewers. Any product that may be evaluated in this article, or claim that may be made by its manufacturer, is not guaranteed or endorsed by the publisher.
