# Supplementary material for: Targeted Next-Generation Sequencing Indicates a Frequent Oligogenic Involvement in Primary Ovarian Insufficiency Onset
Source: Front Endocrinol (Lausanne). 2021 Nov 4;12:664645. doi: 10.3389/fendo.2021.664645 (PMC8600266; doi:10.3389/fendo.2021.664645)
Supplement: Supplementary file 1 [file Table_1.docx]

Supplementary Material

**Table S1. POI gene list analyzed by NGS. The table has been obtained by interrogating the Multi-symbol Checker tool (HGNC).**

| **Gene** | **Gene name** | **HGNC ID** | **Location** |
| --- | --- | --- | --- |
| ADAMTS16 | ADAM metallopeptidase with thrombospondin type 1 motif 16 | HGNC:17108 | 5p15.32 |
| ADAMTS4 | ADAM metallopeptidase with thrombospondin type 1 motif 4 | HGNC:220 | 1q23.3 |
| ADAMTS5 | ADAM metallopeptidase with thrombospondin type 1 motif 5 | HGNC:221 | 21q21.3 |
| ADIPOR1 | adiponectin receptor 1 | HGNC:24040 | 1q32.1 |
| AFP | alpha fetoprotein | HGNC:317 | 4q13.3 |
| AGO1 | argonaute RISC component 1 | HGNC:3262 | 1p34.3 |
| AGRN | agrin | HGNC:329 | 1p36.33 |
| AJUBA | ajuba LIM protein | HGNC:20250 | 14q11.2 |
| AKAP9 | A-kinase anchoring protein 9 | HGNC:379 | 7q21.2 |
| AKT2 | AKT serine/threonine kinase 2 | HGNC:392 | 19q13.2 |
| AMH | anti-Mullerian hormone | HGNC:464 | 19p13.3 |
| AMHR2 | anti-Mullerian hormone receptor type 2 | HGNC:465 | 12q13.13 |
| ANAPC1 | anaphase promoting complex subunit 1 | HGNC:19988 | 2q13 |
| ANAPC2 | anaphase promoting complex subunit 2 | HGNC:19989 | 9q34.3 |
| APAF1 | apoptotic peptidase activating factor 1 | HGNC:576 | 12q23.1 |
| APBB1IP | amyloid beta precursor protein binding family B member 1 interacting protein | HGNC:17379 | 10p12.1 |
| APC | APC regulator of WNT signaling pathway | HGNC:583 | 5q22.2 |
| APC2 | APC regulator of WNT signaling pathway 2 | HGNC:24036 | 19p13.3 |
| AR | androgen receptor | HGNC:644 | Xq12 |
| ARHGEF7 | Rho guanine nucleotide exchange factor 7 | HGNC:15607 | 13q34 |
| ATG2A | autophagy related 2A | HGNC:29028 | 11q13.1 |
| ATG4B | autophagy related 4B cysteine peptidase | HGNC:20790 | 2q37.3 |
| ATG4C | autophagy related 4C cysteine peptidase | HGNC:16040 | 1p31.3 |
| ATG9B | autophagy related 9B | HGNC:21899 | 7q36.1 |
| ATM | ATM serine/threonine kinase | HGNC:795 | 11q22.3 |
| ATR | ATR serine/threonine kinase | HGNC:882 | 3q23 |
| AURKA | aurora kinase A | HGNC:11393 | 20q13.2 |
| AURKB | aurora kinase B | HGNC:11390 | 17p13.1 |
| B4GALT1 | beta-1,4-galactosyltransferase 1 | HGNC:924 | 9p21.1 |
| BARD1 | BRCA1 associated RING domain 1 | HGNC:952 | 2q35 |
| BCOR | BCL6 corepressor | HGNC:20893 | Xp11.4 |
| BDNF | brain derived neurotrophic factor | HGNC:1033 | 11p14.1 |
| BLM | BLM RecQ like helicase | HGNC:1058 | 15q26.1 |
| BMF | Bcl2 modifying factor | HGNC:24132 | 15q15.1 |
| BMP15 | bone morphogenetic protein 15 | HGNC:1068 | Xp11.22 |
| BMP5 | bone morphogenetic protein 5 | HGNC:1072 | 6p12.1 |
| BMP6 | bone morphogenetic protein 6 | HGNC:1073 | 6p24.3 |
| BMP8B | bone morphogenetic protein 8b | HGNC:1075 | 1p34.2 |
| BMPR2 | bone morphogenetic protein receptor type 2 | HGNC:1078 | 2q33.1-q33.2 |
| BRCA1 | BRCA1 DNA repair associated | HGNC:1100 | 17q21.31 |
| BRCA2 | BRCA2 DNA repair associated | HGNC:1101 | 13q13.1 |
| BRD2 | bromodomain containing 2 | HGNC:1103 | 6p21.32 |
| BRD3 | bromodomain containing 3 | HGNC:1104 | 9q34.2 |
| BRD4 | bromodomain containing 4 | HGNC:13575 | 19p13.12 |
| BRDT | bromodomain testis associated | HGNC:1105 | 1p22.1 |
| BRSK1 | BR serine/threonine kinase 1 | HGNC:18994 | 19q13.42 |
| C15orf60 | REC114 meiotic recombination protein | HGNC:25065 | 15q24.1 |
| CARM1 | coactivator associated arginine methyltransferase 1 | HGNC:23393 | 19p13.2 |
| CBX2 | chromobox 2 | HGNC:1552 | 17q25.3 |
| CCNA2 | cyclin A2 | HGNC:1578 | 4q27 |
| CCNB1IP1 | cyclin B1 interacting protein 1 | HGNC:19437 | 14q11.2 |
| CDC25B | cell division cycle 25B | HGNC:1726 | 20p13 |
| CDK8 | cyclin dependent kinase 8 | HGNC:1779 | 13q12.13 |
| CDK9 | cyclin dependent kinase 9 | HGNC:1780 | 9q34.11 |
| CHEK2 | checkpoint kinase 2 | HGNC:16627 | 22q12.1 |
| CHUK | component of inhibitor of nuclear factor kappa B kinase complex | HGNC:1974 | 10q24.31 |
| CITED2 | Cbp/p300 interacting transactivator with Glu/Asp rich carboxy-terminal domain 2 | HGNC:1987 | 6q24.1 |
| COL6A1 | collagen type VI alpha 1 chain | HGNC:2211 | 21q22.3 |
| COL6A2 | collagen type VI alpha 2 chain | HGNC:2212 | 21q22.3 |
| CYP21A2 | cytochrome P450 family 21 subfamily A member 2 | HGNC:2600 | 6p21.33 |
| DHCR24 | 24-dehydrocholesterol reductase | HGNC:2859 | 1p32.3 |
| DICER1 | dicer 1, ribonuclease III | HGNC:17098 | 14q32.13 |
| DLC1 | DLC1 Rho GTPase activating protein | HGNC:2897 | 8p22 |
| DMC1 | DNA meiotic recombinase 1 | HGNC:2927 | 22q13.1 |
| DMRT3 | doublesex and mab-3 related transcription factor 3 | HGNC:13909 | 9p24.3 |
| DMRTC2 | DMRT like family C2 | HGNC:13911 | 19q13.2 |
| DND1 | DND microRNA-mediated repression inhibitor 1 | HGNC:23799 | 5q31.3 |
| DUSP22 | dual specificity phosphatase 22 | HGNC:16077 | 6p25.3 |
| DUSP26 | dual specificity phosphatase 26 | HGNC:28161 | 8p12 |
| EDNRB | endothelin receptor type B | HGNC:3180 | 13q22.3 |
| EGFR | epidermal growth factor receptor | HGNC:3236 | 7p11.2 |
| EP300 | E1A binding protein p300 | HGNC:3373 | 22q13.2 |
| ERBB3 | erb-b2 receptor tyrosine kinase 3 | HGNC:3431 | 12q13.2 |
| ERBB4 | erb-b2 receptor tyrosine kinase 4 | HGNC:3432 | 2q34 |
| ERCC1 | ERCC excision repair 1, endonuclease non-catalytic subunit | HGNC:3433 | 19q13.32 |
| ESR2 | estrogen receptor 2 | HGNC:3468 | 14q23.2-q23.3 |
| EXO1 | exonuclease 1 | HGNC:3511 | 1q43 |
| EZH2 | enhancer of zeste 2 polycomb repressive complex 2 subunit | HGNC:3527 | 7q36.1 |
| FABP6 | fatty acid binding protein 6 | HGNC:3561 | 5q33.3 |
| FANCA | FA complementation group A | HGNC:3582 | 16q24.3 |
| FANCC | FA complementation group C | HGNC:3584 | 9q22.32 |
| FGF16 | fibroblast growth factor 16 | HGNC:3672 | Xq21.1 |
| FGF19 | fibroblast growth factor 19 | HGNC:3675 | 11q13.3 |
| FGF3 | fibroblast growth factor 3 | HGNC:3681 | 11q13.3 |
| FIGLA | folliculogenesis specific bHLH transcription factor | HGNC:24669 | 2p13.3 |
| FKBP6 | FKBP prolyl isomerase family member 6 (inactive) | HGNC:3722 | 7q11.23 |
| FN1 | fibronectin 1 | HGNC:3778 | 2q35 |
| FOXC1 | forkhead box C1 | HGNC:3800 | 6p25.3 |
| FOXE1 | forkhead box E1 | HGNC:3806 | 9q22.33 |
| FOXL2 | forkhead box L2 | HGNC:1092 | 3q22.3 |
| FOXO1 | forkhead box O1 | HGNC:3819 | 13q14.11 |
| FSHR | follicle stimulating hormone receptor | HGNC:3969 | 2p16.3 |
| FST | follistatin | HGNC:3971 | 5q11.2 |
| FSTL3 | follistatin like 3 | HGNC:3973 | 19p13.3 |
| FZD5 | frizzled class receptor 5 | HGNC:4043 | 2q33.3 |
| GDF9 | growth differentiation factor 9 | HGNC:4224 | 5q31.1 |
| GGT1 | gamma-glutamyltransferase 1 | HGNC:4250 | 22q11.23 |
| GGT5 | gamma-glutamyltransferase 5 | HGNC:4260 | 22q11.23 |
| GLI1 | GLI family zinc finger 1 | HGNC:4317 | 12q13.3 |
| GLI2 | GLI family zinc finger 2 | HGNC:4318 | 2q14.2 |
| GNRHR | gonadotropin releasing hormone receptor | HGNC:4421 | 4q13.2 |
| GPC3 | glypican 3 | HGNC:4451 | Xq26.2 |
| GPR137C | G protein-coupled receptor 137C | HGNC:25445 | 14q22.1 |
| GREM2 | gremlin 2, DAN family BMP antagonist | HGNC:17655 | 1q43 |
| GRIP1 | glutamate receptor interacting protein 1 | HGNC:18708 | 12q14.3 |
| GSK3B | glycogen synthase kinase 3 beta | HGNC:4617 | 3q13.33 |
| H2AFX | H2A.X variant histone | HGNC:4739 | 11q23.3 |
| H3F3A | H3.3 histone A | HGNC:4764 | 1q42.12 |
| HDAC5 | histone deacetylase 5 | HGNC:14068 | 17q21.31 |
| HEY2 | hes related family bHLH transcription factor with YRPW motif 2 | HGNC:4881 | 6q22.31 |
| HFM1 | helicase for meiosis 1 | HGNC:20193 | 1p22.2 |
| HIF3A | hypoxia inducible factor 3 subunit alpha | HGNC:15825 | 19q13.32 |
| HK3 | hexokinase 3 | HGNC:4925 | 5q35.2 |
| HORMAD1 | HORMA domain containing 1 | HGNC:25245 | 1q21.3 |
| HPRT1 | hypoxanthine phosphoribosyltransferase 1 | HGNC:5157 | Xq26.2-q26.3 |
| HSD17B4 | hydroxysteroid 17-beta dehydrogenase 4 | HGNC:5213 | 5q23.1 |
| HSP90AB1 | heat shock protein 90 alpha family class B member 1 | HGNC:5258 | 6p21.1 |
| HUS1 | HUS1 checkpoint clamp component | HGNC:5309 | 7p12.3 |
| ID1 | inhibitor of DNA binding 1, HLH protein | HGNC:5360 | 20q11.21 |
| ID2 | inhibitor of DNA binding 2 | HGNC:5361 | 2p25.1 |
| ID3 | inhibitor of DNA binding 3, HLH protein | HGNC:5362 | 1p36.12 |
| IL6ST | interleukin 6 cytokine family signal transducer | HGNC:6021 | 5q11.2 |
| INHA | inhibin subunit alpha | HGNC:6065 | 2q35 |
| INHBA | inhibin subunit beta A | HGNC:6066 | 7p14.1 |
| ITCH | itchy E3 ubiquitin protein ligase | HGNC:13890 | 20q11.22 |
| JHDM1D | lysine demethylase 7A | HGNC:22224 | 7q34 |
| JUP | junction plakoglobin | HGNC:6207 | 17q21.2 |
| KAT2A | lysine acetyltransferase 2A | HGNC:4201 | 17q21.2 |
| KCNIP3 | potassium voltage-gated channel interacting protein 3 | HGNC:15523 | 2q11.1 |
| KDM6B | lysine demethylase 6B | HGNC:29012 | 17p13.1 |
| KDR | kinase insert domain receptor | HGNC:6307 | 4q12 |
| KPNA2 | karyopherin subunit alpha 2 | HGNC:6395 | 17q24.2 |
| LARS2 | leucyl-tRNA synthetase 2, mitochondrial | HGNC:17095 | 3p21.31 |
| LATS1 | large tumor suppressor kinase 1 | HGNC:6514 | 6q25.1 |
| LGR4 | leucine rich repeat containing G protein-coupled receptor 4 | HGNC:13299 | 11p14.1 |
| LHCGR | luteinizing hormone/choriogonadotropin receptor | HGNC:6585 | 2p16.3 |
| LHX8 | LIM homeobox 8 | HGNC:28838 | 1p31.1 |
| LIG1 | DNA ligase 1 | HGNC:6598 | 19q13.33 |
| LIN28A | lin-28 homolog A | HGNC:15986 | 1p36.11 |
| LOR | loricrin cornified envelope precursor protein | HGNC:6663 | 1q21.3 |
| LRP5 | LDL receptor related protein 5 | HGNC:6697 | 11q13.2 |
| MAP3K4 | mitogen-activated protein kinase kinase kinase 4 | HGNC:6856 | 6q26 |
| MCL1 | MCL1 apoptosis regulator, BCL2 family member | HGNC:6943 | 1q21.2 |
| MCM8 | minichromosome maintenance 8 homologous recombination repair factor | HGNC:16147 | 20p12.3 |
| MCM9 | minichromosome maintenance 9 homologous recombination repair factor | HGNC:21484 | 6q22.31 |
| MCPH1 | microcephalin 1 | HGNC:6954 | 8p23.1 |
| MEI1 | meiotic double-stranded break formation protein 1 | HGNC:28613 | 22q13.2 |
| MEX3B | mex-3 RNA binding family member B | HGNC:25297 | 15q25.2 |
| MLH1 | mutL homolog 1 | HGNC:7127 | 3p22.2 |
| MLH3 | mutL homolog 3 | HGNC:7128 | 14q24.3 |
| MLL2 | lysine methyltransferase 2D | HGNC:7133 | 12q13.12 |
| MMP2 | matrix metallopeptidase 2 | HGNC:7166 | 16q12.2 |
| MND1 | meiotic nuclear divisions 1 | HGNC:24839 | 4q31.3 |
| MOGAT1 | monoacylglycerol O-acyltransferase 1 | HGNC:18210 | 2q36.1 |
| MRE11A | MRE11 homolog, double strand break repair nuclease | HGNC:7230 | 11q21 |
| MSH4 | mutS homolog 4 | HGNC:7327 | 1p31.1 |
| MSH5 | mutS homolog 5 | HGNC:7328 | 6p21.33 |
| MTOR | mechanistic target of rapamycin kinase | HGNC:3942 | 1p36.22 |
| MTRR | 5-methyltetrahydrofolate-homocysteine methyltransferase reductase | HGNC:7473 | 5p15.31 |
| MYL9 | myosin light chain 9 | HGNC:15754 | 20q11.23 |
| NABP2 | nucleic acid binding protein 2 | HGNC:28412 | 12q13.3 |
| NANOS2 | nanos C2HC-type zinc finger 2 | HGNC:23292 | 19q13.32 |
| NBN | nibrin | HGNC:7652 | 8q21.3 |
| NCOA3 | nuclear receptor coactivator 3 | HGNC:7670 | 20q13.12 |
| NCOA6 | nuclear receptor coactivator 6 | HGNC:15936 | 20q11.22 |
| NCOR2 | nuclear receptor corepressor 2 | HGNC:7673 | 12q24.31 |
| NEDD4L | NEDD4 like E3 ubiquitin protein ligase | HGNC:7728 | 18q21.31 |
| NOBOX | NOBOX oogenesis homeobox | HGNC:22448 | 7q35 |
| NOS1 | nitric oxide synthase 1 | HGNC:7872 | 12q24.22 |
| NOS3 | nitric oxide synthase 3 | HGNC:7876 | 7q36.1 |
| NOTCH2 | notch receptor 2 | HGNC:7882 | 1p12 |
| NOTCH3 | notch receptor 3 | HGNC:7883 | 19p13.12 |
| NOTCH4 | notch receptor 4 | HGNC:7884 | 6p21.32 |
| NR1D1 | nuclear receptor subfamily 1 group D member 1 | HGNC:7962 | 17q21.1 |
| NR2C2 | nuclear receptor subfamily 2 group C member 2 | HGNC:7972 | 3p25.1 |
| NR4A1 | nuclear receptor subfamily 4 group A member 1 | HGNC:7980 | 12q13.13 |
| NR5A1 | nuclear receptor subfamily 5 group A member 1 | HGNC:7983 | 9q33.3 |
| NRIP1 | nuclear receptor interacting protein 1 | HGNC:8001 | 21q11.2-q21.1 |
| NUP107 | nucleoporin 107 | HGNC:29914 | 12q15 |
| NUP153 | nucleoporin 153 | HGNC:8062 | 6p22.3 |
| NUP205 | nucleoporin 205 | HGNC:18658 | 7q33 |
| NUP214 | nucleoporin 214 | HGNC:8064 | 9q34.13 |
| PARD3 | par-3 family cell polarity regulator | HGNC:16051 | 10p11.22-p11.21 |
| PAXIP1 | PAX interacting protein 1 | HGNC:8624 | 7q36.2 |
| PCSK5 | proprotein convertase subtilisin/kexin type 5 | HGNC:8747 | 9q21.13 |
| PDE3A | phosphodiesterase 3A | HGNC:8778 | 12p12.2 |
| PDGFRB | platelet derived growth factor receptor beta | HGNC:8804 | 5q32 |
| PIK3CG | phosphatidylinositol-4,5-bisphosphate 3-kinase catalytic subunit gamma | HGNC:8978 | 7q22.3 |
| PKP1 | plakophilin 1 | HGNC:9023 | 1q32.1 |
| PLCG1 | phospholipase C gamma 1 | HGNC:9065 | 20q12 |
| PLEC | plectin | HGNC:9069 | 8q24.3 |
| POLB | DNA polymerase beta | HGNC:9174 | 8p11.21 |
| POLE | DNA polymerase epsilon, catalytic subunit | HGNC:9177 | 12q24.33 |
| POLG | DNA polymerase gamma, catalytic subunit | HGNC:9179 | 15q26.1 |
| POPDC3 | popeye domain containing 3 | HGNC:17649 | 6q21 |
| PRDM9 | PR/SET domain 9 | HGNC:13994 | 5p14.2 |
| PRDX5 | peroxiredoxin 5 | HGNC:9355 | 11q13.1 |
| PRIM1 | DNA primase subunit 1 | HGNC:9369 | 12q13.3 |
| PRKAA2 | protein kinase AMP-activated catalytic subunit alpha 2 | HGNC:9377 | 1p32.2 |
| PRL | prolactin | HGNC:9445 | 6p22.3 |
| PSMC3IP | PSMC3 interacting protein | HGNC:17928 | 17q21.2 |
| PSMC4 | proteasome 26S subunit, ATPase 4 | HGNC:9551 | 19q13.11-q13.13 |
| PSMC5 | proteasome 26S subunit, ATPase 5 | HGNC:9552 | 17q23.3 |
| PSMD4 | proteasome 26S subunit ubiquitin receptor, non-ATPase 4 | HGNC:9561 | 1q21.3 |
| PSMD7 | proteasome 26S subunit, non-ATPase 7 | HGNC:9565 | 16q23.1 |
| PSMD8 | proteasome 26S subunit, non-ATPase 8 | HGNC:9566 | 19q13.2 |
| PTEN | phosphatase and tensin homolog | HGNC:9588 | 10q23.31 |
| PTGER2 | prostaglandin E receptor 2 | HGNC:9594 | 14q22.1 |
| PTGS2 | prostaglandin-endoperoxide synthase 2 | HGNC:9605 | 1q31.1 |
| PTHLH | parathyroid hormone like hormone | HGNC:9607 | 12p11.22 |
| PTK2 | protein tyrosine kinase 2 | HGNC:9611 | 8q24.3 |
| RAD1 | RAD1 checkpoint DNA exonuclease | HGNC:9806 | 5p13.2 |
| RAD21L1 | RAD21 cohesin complex component like 1 | HGNC:16271 | 20p13 |
| RAD50 | RAD50 double strand break repair protein | HGNC:9816 | 5q31.1 |
| RAD51 | RAD51 recombinase | HGNC:9817 | 15q15.1 |
| RAD52 | RAD52 homolog, DNA repair protein | HGNC:9824 | 12p13.33 |
| RAD54L | RAD54 like | HGNC:9826 | 1p34.1 |
| RASAL2 | RAS protein activator like 2 | HGNC:9874 | 1q25.2 |
| RBBP8 | RB binding protein 8, endonuclease | HGNC:9891 | 18q11.2 |
| RBPJ | recombination signal binding protein for immunoglobulin kappa J region | HGNC:5724 | 4p15.2 |
| REC8 | REC8 meiotic recombination protein | HGNC:16879 | 14q12 |
| RELN | reelin | HGNC:9957 | 7q22.1 |
| RFC4 | replication factor C subunit 4 | HGNC:9972 | 3q27.3 |
| RIPK1 | receptor interacting serine/threonine kinase 1 | HGNC:10019 | 6p25.2 |
| RMI1 | RecQ mediated genome instability 1 | HGNC:25764 | 9q21.32 |
| RNF212 | ring finger protein 212 | HGNC:27729 | 4p16.3 |
| RORA | RAR related orphan receptor A | HGNC:10258 | 15q22.2 |
| RPA1 | replication protein A1 | HGNC:10289 | 17p13.3 |
| RPN2 | ribophorin II | HGNC:10382 | 20q11.23 |
| RYR3 | ryanodine receptor 3 | HGNC:10485 | 15q13.3-q14 |
| SAMD11 | sterile alpha motif domain containing 11 | HGNC:28706 | 1p36.33 |
| SEL1L | SEL1L adaptor subunit of ERAD E3 ubiquitin ligase | HGNC:10717 | 14q31 |
| SETD8 | lysine methyltransferase 5A | HGNC:29489 | 12q24.31 |
| SIGLEC11 | sialic acid binding Ig like lectin 11 | HGNC:15622 | 19q13.33 |
| SIRT1 | sirtuin 1 | HGNC:14929 | 10q21.3 |
| SKI | SKI proto-oncogene | HGNC:10896 | 1p36.33-p36.32 |
| SMAD5 | SMAD family member 5 | HGNC:6771 | 5q31.1 |
| SMAD6 | SMAD family member 6 | HGNC:6772 | 15q22.31 |
| SMAD7 | SMAD family member 7 | HGNC:6773 | 18q21.1 |
| SMARCA4 | SWI/SNF related, matrix associated, actin dependent regulator of chromatin, subfamily a, member 4 | HGNC:11100 | 19p13.2 |
| SMC1B | structural maintenance of chromosomes 1B | HGNC:11112 | 22q13.31 |
| SMURF2 | SMAD specific E3 ubiquitin protein ligase 2 | HGNC:16809 | 17q23.3-q24.1 |
| SNRNP48 | small nuclear ribonucleoprotein U11/U12 subunit 48 | HGNC:21368 | 6p24.3 |
| SOX8 | SRY-box transcription factor 8 | HGNC:11203 | 16p13.3 |
| SPATA22 | spermatogenesis associated 22 | HGNC:30705 | 17p13.2 |
| SPO11 | SPO11 initiator of meiotic double stranded breaks | HGNC:11250 | 20q13.31 |
| SSTR2 | somatostatin receptor 2 | HGNC:11331 | 17q25.1 |
| STAG1 | stromal antigen 1 | HGNC:11354 | 3q22.3 |
| STAG3 | stromal antigen 3 | HGNC:11356 | 7q22.1 |
| STAR | steroidogenic acute regulatory protein | HGNC:11359 | 8p11.23 |
| SUV420H2 | lysine methyltransferase 5C | HGNC:28405 | 19q13.42 |
| SYCE1 | synaptonemal complex central element protein 1 | HGNC:28852 | 10q26.3 |
| SYCE2 | synaptonemal complex central element protein 2 | HGNC:27411 | 19p13.13 |
| SYCE3 | synaptonemal complex central element protein 3 | HGNC:35245 | 22q13.33 |
| SYCP1 | synaptonemal complex protein 1 | HGNC:11487 | 1p13.2 |
| SYCP2 | synaptonemal complex protein 2 | HGNC:11490 | 20q13.33 |
| SYCP2L | synaptonemal complex protein 2 like | HGNC:21537 | 6p24.2 |
| SYCP3 | synaptonemal complex protein 3 | HGNC:18130 | 12q23.2 |
| SYNE1 | spectrin repeat containing nuclear envelope protein 1 | HGNC:17089 | 6q25.2 |
| SYNE2 | spectrin repeat containing nuclear envelope protein 2 | HGNC:17084 | 14q23.2 |
| SYNGAP1 | synaptic Ras GTPase activating protein 1 | HGNC:11497 | 6p21.32 |
| SYVN1 | synoviolin 1 | HGNC:20738 | 11q13.1 |
| TAF4B | TATA-box binding protein associated factor 4b | HGNC:11538 | 18q11.2 |
| TBP | TATA-box binding protein | HGNC:11588 | 6q27 |
| TBX3 | T-box transcription factor 3 | HGNC:11602 | 12q24.21 |
| TERT | telomerase reverse transcriptase | HGNC:11730 | 5p15.33 |
| TEX11 | testis expressed 11 | HGNC:11733 | Xp11 |
| TEX12 | testis expressed 12 | HGNC:11734 | 11q23.1 |
| TEX15 | testis expressed 15, meiosis and synapsis associated | HGNC:11738 | 8p12 |
| THBS2 | thrombospondin 2 | HGNC:11786 | 6q27 |
| TJP2 | tight junction protein 2 | HGNC:11828 | 9q21.11 |
| TLN1 | talin 1 | HGNC:11845 | 9p13.3 |
| TNFAIP6 | TNF alpha induced protein 6 | HGNC:11898 | 2q23.3 |
| TOP3A | DNA topoisomerase III alpha | HGNC:11992 | 17p11.2 |
| TOP3B | DNA topoisomerase III beta | HGNC:11993 | 22q11.22 |
| TOPAZ1 | testis and ovary specific TOPAZ 1 | HGNC:24746 | 3p21.31 |
| TOPBP1 | DNA topoisomerase II binding protein 1 | HGNC:17008 | 3q22.1 |
| TP53 | tumor protein p53 | HGNC:11998 | 17p13.1 |
| TP63 | tumor protein p63 | HGNC:15979 | 3q28 |
| TP73 | tumor protein p73 | HGNC:12003 | 1p36.32 |
| TRRAP | transformation/transcription domain associated protein | HGNC:12347 | 7q22.1 |
| TSC1 | TSC complex subunit 1 | HGNC:12362 | 9q34 |
| TUBA8 | tubulin alpha 8 | HGNC:12410 | 22q11.21 |
| TUBB4B | tubulin beta 4B class IVb | HGNC:20771 | 9q34.3 |
| UBR2 | ubiquitin protein ligase E3 component n-recognin 2 | HGNC:21289 | 6p21.1 |
| UMODL1 | uromodulin like 1 | HGNC:12560 | 21q22.3 |
| USP35 | ubiquitin specific peptidase 35 | HGNC:20061 | 11q14.1 |
| VLDLR | very low density lipoprotein receptor | HGNC:12698 | 9p24.2 |
| VWF | von Willebrand factor | HGNC:12726 | 12p13.31 |
| WNT2 | Wnt family member 2 | HGNC:12780 | 7q31.2 |
| XIAP | X-linked inhibitor of apoptosis | HGNC:592 | Xq25 |
| YAP1 | Yes1 associated transcriptional regulator | HGNC:16262 | 11q22.1 |
| YBX2 | Y-box binding protein 2 | HGNC:17948 | 17p13.1 |
| YWHAQ | tyrosine 3-monooxygenase/tryptophan 5-monooxygenase activation protein theta | HGNC:12854 | 2p25.1 |
| YY1 | YY1 transcription factor | HGNC:12856 | 14q32.2 |
