# Supplementary material for: Targeted Next-Generation Sequencing Indicates a Frequent Oligogenic Involvement in Primary Ovarian Insufficiency Onset
Source: Front Endocrinol (Lausanne). 2021 Nov 4;12:664645. doi: 10.3389/fendo.2021.664645 (PMC8600266; doi:10.3389/fendo.2021.664645)
Supplement: Supplementary file 2 [file Table_2.docx]

Supplementary Material

**Table S2. Summary of the candidate gene variations and *in-silico* predictions found in 64 patients analyzed on the *OVO-Array* panel.** Frequencies of the variants in the POI group and in the female population are reported for each gene variant, together with VarSome prediction. In the “VarSome Pathogenicity Scores” column the damaging, disease-causing or deleterious predictions are red colored, benign or tolerated predictions are displayed in green, while the unknown predictions are represented in orange. The Genomic Evolutionary Rate Profiling (GERP) is a conservation score calculated by quantifying substitution deficits across multiple alignments of orthologues using the genomes of 35 mammals. It ranges from -12.3 to 6.17, with 6.17 being the most conserved (Cooper *et al.*, 2005). GERP NR: 'neutral rate' score of the site VarSome; GERP RS: GERP typical score which quantifies position-specific constraint in terms of rejected substitutions (RS) by estimating the actual number of substitutions at that site and subtracting it from the number expected assuming neutrality. Genes and variants already associated to POI are red and green colored, respectively.

| **Pathway** | | **Gene** | **Transcript** | **cDNA Variation** | **Protein Variation** | **OVO-Array patients’ frequency(n=64)** | **gnomAD ver. 2.1.1 female population frequency** | **VarSome Pathogenicity Scores** | **Link to VarSome** | **references** |
| --- | --- | --- | --- | --- | --- | --- | --- | --- | --- | --- |
| CELL CYCLE  MEIOSIS  TP53/DNA REPAIR | | *ANAPC1* | NM_022662 | c.2279C>G | p.Pro760Arg | 0.015625 | 0.0000607 | 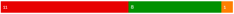 | varso.me/Nd2G | Grøndahl *et al*., 2010 |
|  |  | *APC2* | NM_005883 | c.2887C>T | p.Arg963Trp | 0.015625 | 0.0000135 | 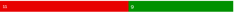 | varso.me/TdbK | Mohamed *et al*., 2019 |
|  |  |  |  | c.932C>T | p.Ser311Leu | 0.015625 | - | 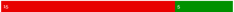 | varso.me/Tdbc |  |
|  |  | *ATM* | NM_000051 | c.418G>C | p.Asp140His | 0.015625 | - | 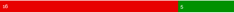 | varso.me/LMjL | Liu *et al*., 2020  França *et al*.,2020 |
|  |  |  |  | c.1444A>C | p.Lys482Gln | 0.015625 | 0.000104 | 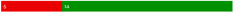 | varso.me/FFvg |  |
|  |  |  |  | c.4829G>C | p.Arg1610Thr | 0.015625 | Novel | 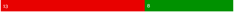 | varso.me/T0Bg |  |
|  |  |  |  | c.7375C>T | p.Arg2459Cys | 0.015625 | 0.0000433 | 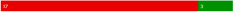 | varso.me/SIfh |  |
|  |  | *ATR* | NM_001184 | c.4610T>A | p.Leu1537* | 0.015625 | Novel | 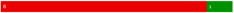 | varso.me/TZxD | Pacheco *et al*., 2018 |
|  |  |  |  | c.2783A>G | p.Gln928Arg | 0.015625 | Novel | 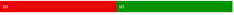 | varso.me/TZxU |  |
|  |  | *BLM* | NM_000057 | c.2333C>G | p.Ser778Cys | 0.015625 | 0.000156 | 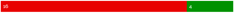 | varso.me/N9MX | Ellis *et al*., 1996; Jiao *et al*., 2018 |
|  |  | *BRCA1* | NM_007294 | c.902A>G | p.Lys301Arg | 0.015625 | Novel | 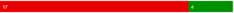 | varso.me/N8By | Porcu *et al*., 2019 |
|  |  | *CCNB1IP1* | NM_182852 | c.454G>A | p.Glu152Lys | 0.015625 | Novel | 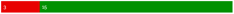 | varso.me/NdyX | Strong *et al*., 2010 |
|  |  | *CHEK2* | NM_007194 | c.1039G>A | Asp347Asn | 0.015625 | 0.0000259 | 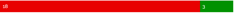 | varso.me/N8CQ | Mustofa et al., 2020 |
|  |  | *FANCA* | NM_000135 | c.1340C>T | p.Ser447Leu | 0.015625 | 0.000486 | 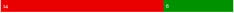 | varso.me/NeiI | Yang *et al*., 2019 |
|  |  | *HDAC5* | NM_001015053 | c.446A>G | p.Glu149Gly | 0.015625 | - | 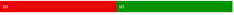 | varso.me/N9pQ | Peixoto *et al*., 2012 |
|  |  | *KMT5A* | NM_020382 | c.287_289delAAG | p.Glu97del | 0.015625 | - | GERP NR 5.1239 (m.n.)  GERP RS 3.6132 (m.n.) | varso.me/N8kY | Cui *et al*., 2017 |
|  |  | *KPNA2* | NM_002266 | c.445T>C | p.Ser149Pro | 0.015625 | Novel | 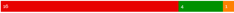 | varso.me/NeQi | Huang *et al*., 2013 |
|  |  | *MCM9* | NM_017696 | c.970G>T | p.Val324Leu | 0.015625 | Novel | 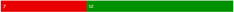 | varso.me/Ncwj | Wood-Trageser *et al*., 2014; Desai *et al*.,2016 |
|  |  | *MLH3* | NM_001040108 | c.3466G>A | p.Val1156Ile | 0.015625 | 0.0000696 | 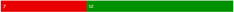 | varso.me/SR8H | Patiño *et al*., 2017; Castéra *et al*., 2014 |
|  |  |  |  | c.3943G>A | p.Glu1315Lys | 0.015625 | - | 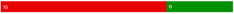 | varso.me/TjgS |  |
|  |  | *MSH4* | NM_002440 | c.1286A>T | p.Glu429Val | 0.015625 | Novel | 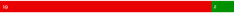 | varso.me/Nd2q | Carlosama *et al*., 2017 |
|  |  | *NBN* | NM_002485 | c.596C>G | p.Pro199Arg | 0.015625 | Novel | 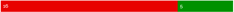 | varso.me/NeTA | França *et al*., 2020; Tucker *et al*., 2018 |
|  |  | *NCOA6* | NM_014071 | c.1250C>G | p.Pro417Arg | 0.015625 | Novel | 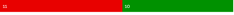 | varso.me/Ndlr | Mahajan *et al*., 2008 |
|  |  | *NCOR2* | NM_006312 | c.3755C>A | p.Pro1252Gln | 0.015625 | Novel | 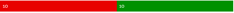 | varso.me/T2Av | Hussein-Fikre *et al*., 2005; Salvetti *et al*., 2012 |
|  |  |  |  | c.3709G>A | p.Val1237Ile | 0.015625 | 0.00000886 | 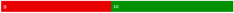 | varso.me/T2BI |  |
|  |  |  |  | c.3760C>T | p.Arg1254Cys | 0.015625 | 0.000123 | 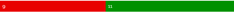 | varso.me/T2BR |  |
|  |  | *PLEC* | NM_201380 | c.5801G>A | p.Arg1934His | 0.015625 | 0.000128 | 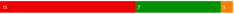 | varso.me/N9kA | Gostyńska *et al*.,2015 |
|  |  | *POLE* | NM_006231 | c.4900C>T | p.Arg1634Cys | 0.015625 | 0.00000868 | 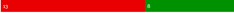 | varso.me/N9Gb | Zegerman 2013; Wimmer *et al*., 2017 |
|  |  | *PRIM1* | NM_000946 | c.911G>T | p.Arg304Leu | 0.015625 | Novel | 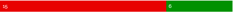 | varso.me/Nacq | Stolk *et al*., 2012 |
|  |  | *RAD50* | NM_005732 | c.2165dupA | p.Glu723Glyfs*5 | 0.015625 | 0.000301 | GERP NR 6.0582 (m.n.)  GERP RS 4.0478 (m.n.) | varso.me/InOI | Roset *et al*., 2014 |
|  |  | *RAD52* | NM_134424 | c.175G>A | p.Gly59Arg | 0.015625 | 0.000699 | 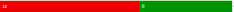 | varso.me/Td7L | Ma *et al*., 2019 |
|  |  |  |  | c.388G>A | p.Glu130Lys | 0.015625 | 0.000138 | 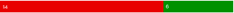 | varso.me/Td7m |  |
|  |  |  |  | c.761C>T | p.Thr254Met | 0.015625 | 0.000149 | 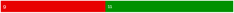 | varso.me/Td7a |  |
|  |  | *RAD54L* | NM_003579 | c.604C>T | p.Arg202Cys | 0.015625 | 0.00324 | 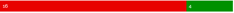 | varso.me/FIC4 | Messiaen *et al*., 2013 |
|  |  |  |  | c.2209C>A | p.Gln737Lys | 0.015625 | Novel | 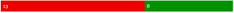 | varso.me/TZvx |  |
|  |  | *RBBP8* | NM_002894 | c.2516G>A | p.Arg839Gln | 0.03125 | 0.000415 | 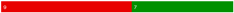 | varso.me/Fdvl | Ledig *et al.,* 2010 |
|  |  | *REC8* | NM_005132 | c.899G>T | p.Arg300Leu | 0.015625 | 0.00234 | 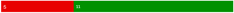 | varso.me/N98t | Bouilly *et al*., 2016; Caburet *et al*., 2014 |
|  |  | *RMI1* | NM_024945 | c.746C>T | p.Ala249Val | 0.015625 | Novel | 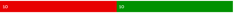 | varso.me/Nac1 | Luo *et al*., 2020 |
|  |  | *STAG3* | NM_012447 | c.1079G>A | p.Arg360His | 0.015625 | - | 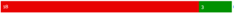 | varso.me/SWXv | Xiao *et al*., 2009 |
|  |  | *SYNE1* | NM_182961 | c.16709A>G | p.Gln5570Arg | 0.015625 | Novel | 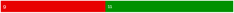 | varso.me/Tdt7 | Baumann *et al*., 2016 |
|  |  | *TEX15* | NM_001350162 | c.6511C>T | p.Arg2171Ter | 0.015625 | 0.0000175 | 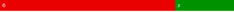 | varso.me/NeXx | Okutman *et al*., 2015 |
|  |  | *TP53* | NM_001126114 | c.475G>A | p.Ala159Thr | 0.015625 | - | 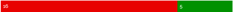 | varso.me/NeTW | Hu *et al*., 2019; Kang *et al*., 2018 |
|  |  | *TP63* | NM_003722 | c.1927C>T | p.Arg643* | 0.015625 | Novel | 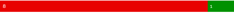 | varso.me/Neir | Tucker *et al*., 2019; Mathorne *et al.*, 2020 |
|  |  | *TP73* | NM_005427 | c.1660T>C | p.Tyr554His | 0.015625 | - | 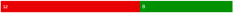 | varso.me/N9v8 | Kang et al., 2018 |
|  |  | *TRRAP* | NM_001244580 | c.10171A>G | p.Met3391Val | 0.015625 | Novel | 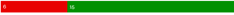 | varso.me/Nc7l | Kang et al., 2018 |
| REPRODUCTION  (folliculogenesis, oocyte maturation and follicular development) | | *AR* | NM_000044 | c.2395C>G | p.Gln799Glu | 0.015625 | 0.00147 | 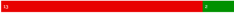 | varso.me/N9xd | Panda et al., 2010 |
|  |  | *ATG2A* | NM_015104 | c.4414G>C | p.Gly1472Arg | 0.015625 | 0.0000174 | 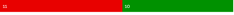 | varso.me/NeQY | Dikic *et al*., 2018 |
|  |  | *ATG4C* | NM_032852 | c.607dupT | p.Trp203Leufs*4 | 0.015625 | 0.000659 | GERP NR 5.92 (m.n.)  GERP RS 4.6425 (m.n.) | varso.me/T0VM |  |
|  |  |  |  | c.774_777delTTAT | p.Ile258Metfs*13 | 0.015625 | 0.0000546 | GERP NR 5.84 (m.n.)  GERP RS 2.3137 (m.n.) | varso.me/T0Vd |  |
|  |  | *BMP15* | NM_005448 | c.202C>T | p.Arg68Trp | 0.015625 | 0.000708 | 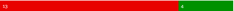 | varso.me/Ne2w | Rossetti *et al*., 2009; Patiño *et al*., 2017 |
|  |  | *CYP21A2* | NM_000500 | c.844G>T | p.Val282Leu | 0.015625 | no data available | no data available | varso.me/N9iB | Speiser *et al*, 1988; Owerbach *et al.*, 1992 |
|  |  |  |  | c.1360C>T | p.Pro454Ser | 0.015625 | no data available | no data available | varso.me/Jl3r |  |
|  |  | *DMRT3* | NM_021240 | c.897dupC | p.Ala300Argfs*4 | 0.015625 | Novel | GERP NR 4.5425 (m.n.)  GERP RS -3.7283 (m.n.) | varso.me/Ne5a | Ottolenghi *et al*., 2000 |
|  |  | *FIGLA* | NM_001004311 | c.364delG | p.Glu122Lysfs*45 | 0.015625 | - | GERP NR 5.56 (m.n.)  GERP RS 4.7349 (m.n.) | varso.me/NejC | Cattoni *et al*., 2020 |
|  |  | *FSHR* | NM_000145 | c.491C>T | p.Ser164Phe | 0.015625 | Novel | 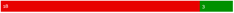 | varso.me/T1wg | Aittomäki *et al*., 1995; Latronico *et al*., 1996; Cordts *et al.,* 2013 |
|  |  |  |  | c.847C>T | p.Arg283Trp | 0.015625 | 0.000052 | 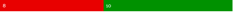 | varso.me/SWBI |  |
|  |  | *GDF9* | NM_005260 | c.278A>G | p.Tyr93Cys | 0.015625 | Novel | 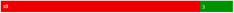 | varso.me/Tjdt | Bouilly *et al*., 2016; Palmer *et al*., 2006; França *et al*., 2017 |
|  |  |  |  | c.1121C>T | p.Pro374Leu | 0.015625 | 0.0000346 | 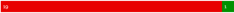 | varso.me/Tjdi |  |
|  |  | *ID1* | NM_002165 | c.458_460delTGT | p.Leu153del | 0.015625 | 0.00000886 | GERP NR 4.76 (m.n.)  GERP RS 4.1775 (m.n.) | varso.me/Rkuo | Li *et al*., 2016 |
|  |  | *KMT2D* | NM_003482 | c.10876C>T | p.Arg3626Trp | 0.015625 | - | 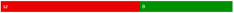 | varso.me/N8Is | Lin *et al*., 2014 |
|  |  | *LHCGR* | NM_000233 | c.568C>A | p.Gln190Lys | 0.015625 | 0.000563 | 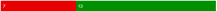 | varso.me/P0M2 | Arnhold *et al*., 2009; Zielen *et al*., 2018 |
|  |  | *NOBOX* | NM_001080413 | c.1626delC | p.Phe543Serfs*7 | 0.015625 | - | GERP NR 4.3499 (m.n.)  GERP RS 2.4529 (m.n.) | varso.me/T1kg | Cattoni *et al*., 2020; Bouilly *et al*., 2015; Bouilly *et al*., 2016; Ferrari *et al*., 2016 |
|  |  |  |  | c.1112A>C | p.Lys371Thr | 0.015625 | 0.000238 | 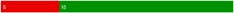 | varso.me/SWXS |  |
|  |  | *NR5A1* | NM_004959 | c.1063G>A | p.Val355Met | 0.03125 | 0.000122 | 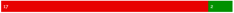 | varso.me/TdLE | Cattoni *et al*., 2020; Philibert *et al*., 2007 |
|  |  |  |  | c.502G>C | p.Ala168Pro | 0.015625 | - | 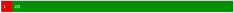 | varso.me/TdIj |  |
|  |  | *TUBA8* | NM_018943 | c.967G>A | p.Val323Met | 0.015625 | 0.000243 | 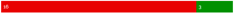 | varso.me/NbX9 | Diggle *et al*., 2013 |
| NOTCH  SIGNALING  (granulosa cell differentiation/ proliferation) | | *NOTCH2* | NM_024408 | c.2084C>A | p.Ala695Glu | 0.015625 | Novel | 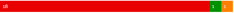 | varso.me/NcAq | Patiño *et al*., 2017; Terauchi *et al*., 2016; Sun *et al*., 2016 |
|  |  | *NOTCH3* | NM_000435 | c.2791A>G | p.Ser931Gly | 0.015625 | Novel | 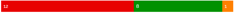 | varso.me/Td99 |  |
|  |  | *NOTCH4* | NM_004557 | c.2945C>T | p.Thr982Ile | 0.015625 | Novel | 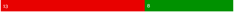 | varso.me/NacO |  |
| CELL METABOLISM | Mitochondrial DNA metabolic process | *POLG* | NM_001126131 | c.752C>T | p.Thr251Ile | 0.03125 | 0.00147 | 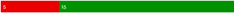 | varso.me/FCE6 | Pagnamenta *et al*., 2006; Luoma P *et al*., 2017; DeBlasi *et al*., 2017; Di Fonzo *et al*., 2003; González-Vioque *et al*.,2006 |
|  |  |  |  | c.803G>C | p.Gly268Ala | 0.015625 | 0.00339 | 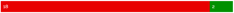 | varso.me/IbzD |  |
|  |  |  |  | c.1760C>T | p.Pro587Leu | 0.03125 | 0.0015 | 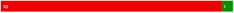 | varso.me/Ez4d |  |
|  |  |  |  | c.3436C>T | p.Arg1146Cys | 0.015625 | 0.000182 | 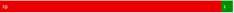 | varso.me/N8U8 |  |
|  | cholesterol synthesis | *DHCR24* | NM_014762 | c.1046C>G | p.Pro349Arg | 0.015625 | 0.0000087 | 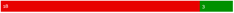 | varso.me/NdwP | Waterham *et al*., 2011 |
|  | glucose methabolism | *HK3* | NM_002115 | c.2077A>C | p.Met693Leu | 0.015625 | 0.000165 | 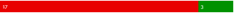 | varso.me/TjkN | Patiño *et al*., 2017 |
|  |  |  |  | c.2389G>A | p.Glu797Lys | 0.015625 | 0.000113 | 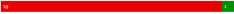 | varso.me/TjkG |  |
|  | aminoacyl-tRNA synthetase | *LARS2* | NM_015340 | c.1021T>G | p.Cys341Gly | 0.015625 | Novel | 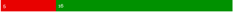 | varso.me/TdCz | Pierce *et al*., 2013; Carminho-Rodrigues et al., 2020 |
|  |  |  |  | c.1717T>C | p.Phe573Leu | 0.015625 | Novel | 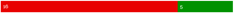 | varso.me/TdEN |  |
|  |  |  |  | c.2192A>T | p.Tyr731Phe | 0.015625 | Novel | 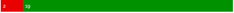 | varso.me/TdGx |  |
|  |  |  |  | c.457A>C | p.Asn153His | 0.015625 | Novel | 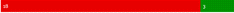 | varso.me/R5je |  |
|  | steroido-genesis | *VLDLR* | NM_003383 | c.902G>A | 0.015625 | 0.015625~~3846~~ | 0.00192 | 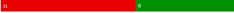 | varso.me/Rko9 | Hussain 2001 |
| ECM  REMODELING | | *ADAMTS4* | NM_005099 | c.803G>A | p.Arg268Gln | 0.015625 | - | 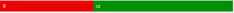 | varso.me/Ndnn | Rogerson *et al*., 2008 |
|  |  | *ADAMTS5* | NM_007038 | c.1729G>A | p.Gly577Ser | 0.03125 | 0.0151 | 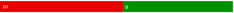 | varso.me/RpWE |  |
|  |  | *ADAMTS16* | NM_139056 | c.2459G>A | p.Arg820Gln | 0.015625 | 0.0000175 | 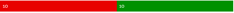 | varso.me/N92c | Patiño *et al*., 2017 |
|  |  | *AGRN* | NM_198576 | c.2860G>A | p.Ala954Thr | 0.015625 | 0.000026 | 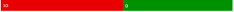 | varso.me/NbqC | Nicole *et al*., 2014 |
|  |  | *COL6A1* | NM_001848 | c.350T>C | p.Val117Ala | 0.015625 | 0.000982 | 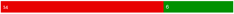 | varso.me/N8Cc | Bovolenta *et al*., 2010 |
|  |  | *COL6A2* | NM_001849 | c.343C>T | p.Arg115Trp | 0.015625 | 0.00000873 | 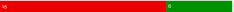 | varso.me/N8DA |  |
|  |  |  |  | c.511G>A | p.Gly171Arg | 0.015625 | 0.00114 | 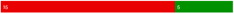 | varso.me/Orla |  |
|  |  |  |  | c.2308G>C | p.Glu770Gln | 0.015625 | Novel | 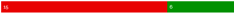 | varso.me/TZnl |  |
|  |  |  |  | c.2575G>A | p.Val859Met | 0.015625 | 0.000241 | GERP NR 4.1799 (m.n.)  GERP RS 3.2999 (m.n.) | varso.me/ReIP |  |
|  |  | *ERBB3* | NM_001982 | c.2269dupA | p.Thr757Asnfs*70 | 0.015625 | Novel | GERP NR 5.3899 (m.n.)  GERP RS 4.3899 (m.n.) | varso.me/N9Gt | Wang *et al*., 2019 |
|  |  | *ERBB4* | NM_005235 | p.Thr32Met | p.Val1115Glu | 0.015625 | Novel | 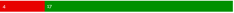 | varso.me/T1xM |  |
|  |  |  |  | c.95C>T | p.Thr32Met | 0.015625 | - | 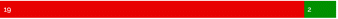 | varso.me/T1x2 |  |
|  |  | *PKP1* | NM_000299 | c.2096A>T | p.Lys699Met | 0.015625 | 0.000156 | 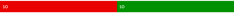 | varso.me/T2Ao | Boyce *et al*., 2011 |
|  |  |  |  | c.883C>G | p.Leu295Val | 0.015625 | 0.00014 | 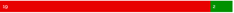 | varso.me/T2AC |  |
|  |  | *RELN* | NM_005045 | c.2015C>T | p.Pro672Leu | 0.015625 | 0.000139 | 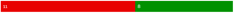 | varso.me/GFVk | Dazzo *et al*., 2015 |
|  |  |  |  | c.3651C>G | p.Ile1217Met | 0.015625 | 0.00255 | 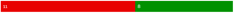 | varso.me/TCd9 |  |
|  |  | *THBS2* | NM_003247 | c.1183G>A | p.Val395Met | 0.015625 | 0.0000528 | 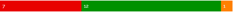 | varso.me/Nd2Z | Noh *et al*., 2003 |
|  |  | *VWF* | NM_000552 | c.4508T>C | p.Leu1503Pro | 0.03125 | Novel | 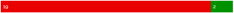 | varso.me/TYo1 | Pérez-Casal *et al*., 1993; Michiels *et al*., 2009 |
|  |  |  |  | c.4517C>T | p.Ser1506Leu | 0.046875 | - | 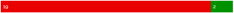 | varso.me/TYnR |  |
|  |  |  |  | c.5641G>A | p.Asp1881Asn | 0.015625 | Novel | 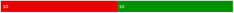 | varso.me/T1dI |  |
| CELL PROLIFERATION | | *SAMD11* | NM_152486 | c.628C>T | p.Arg210Cys | 0.015625 | 0.000536 | 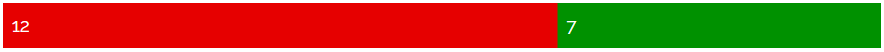 | varso.me/N60l | Inoue *et al.* 2006; Jin *et al*. 2013 |
|  |  |  |  | c.682_683insT | p.Pro228Leufs*227 | 0.09375 | 0.000183 | GERP NR 1.2799 (m.n.)  GERP RS 0.6534 (m.n.) | varso.me/N7qi |  |
| CELL DEATH | | *RIPK1* | NM_003804 | c.700G>A | p.Glu234Lys | 0.015625 | 0.000684 | 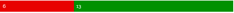 | varso.me/Nd2P | Yue L *et al*., 2019 |
| WNT  SIGNALING | | *LGR4* | NM_018490 | c.2531A>G | p.Asp844Gly | 0.015625 | 0.0124 | 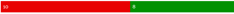 | varso.me/TZmC | Styrkarsdottir *et al*., 2013; Glinka *et al.*, 2011 |
|  |  | *LRP5* | NM_002335 | c.4511C>T | p.Pro1504Leu | 0.015625 | 0.000389 | 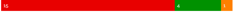 | varso.me/NeTn | Grünblatt *et al*., 2018; Kamiński *et al*., 2019 |
| UBIQUITINATION | | *USP35* | NM_020798 | c.1963dup | p.Thr655Asnfs*74 | 0.015625 | 0.0000267 | GERP NR 4.86 (m.n.)  GERP RS -4.73 (m.n.) | varso.me/N6iM | Park *et al*., 2018 |
| SIGNAL TRANSDUCTION | | *AKAP9* | NM_005751 | c.4351A>G | p.Met1451Val | 0.015625 | - | 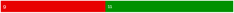 | varso.me/N9oA | Schimenti *et al*., 2013 |
|  |  | *GPR137C* | NM_001099652 | c.1211A>G | p.Asp404Gly | 0.015625 | Novel | 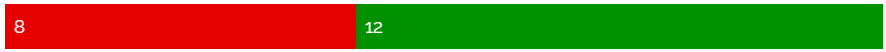 | varso.me/N7n2 | Gan *et al*., 2019 |
|  |  | *RASAL2* | NM_004841 | c.3187G>A | p.Glu1063Lys | 0.015625 | 0.000338 | 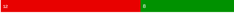 | varso.me/N8xZ | Huang *et al*., 2014 |
| CALCIUM RELEASE | | *RYR3* | NM_001036 | c.393C>A | p.Asp131Glu | 0.015625 | - | 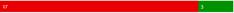 | varso.me/T1hi | Dulhunty *et al*., 2018 |
|  |  |  |  | c.592A>G | p.Met198Val | 0.015625 | - | 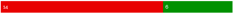 | varso.me/T1gq |  |
|  |  |  |  | c.14584C>T | p.Arg4862Cys | 0.015625 | 0.0000175 | 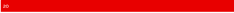 | varso.me/T1fv |  |
|  |  |  |  | c.13709 G>A | p.Arg4570His | 0.015625 | 0.00000877 | 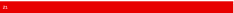 | varso.me/T1hS |  |

**References**

Aittomäki K, Lucena JD, Pakarinen P, Sistonen P, Tapanainen J, Gromoll J, Kaskikari R, Sankila E-M, Lehväslaiho H, Engel AR, et al. Mutation in the follicle-stimulating hormone receptor gene causes hereditary hypergonadotropic ovarian failure. Cell (1995) 82:959–968. doi:10.1016/0092-8674(95)90275-9

Arnhold IJ, Lofrano-Porto A, Latronico AC. Inactivating mutations of luteinizing hormone beta-subunit or luteinizing hormone receptor cause oligo-amenorrhea and infertility in women. Horm Res. 2009;71(2):75–82

Baumann M, Steichen-Gersdorf E, Krabichler B, Petersen B-S, Weber U, Schmidt WM, Zschocke J, Müller T, Bittner RE, Janecke AR. Homozygous SYNE1 mutation causes congenital onset of muscular weakness with distal arthrogryposis: a genotype–phenotype correlation. European Journal of Human Genetics (2016) 25:262–266. doi:10.1038/ejhg.2016.144

Bouilly J, Roucher-Boulez F, Gompel A, Bry-Gauillard H, Azibi K, Beldjord C, Dodé C, Bouligand J, Mantel AG, Hécart A-C, et al. New NOBOX Mutations Identified in a Large Cohort of Women With Primary Ovarian Insufficiency Decrease KIT-L Expression. The Journal of Clinical Endocrinology & Metabolism (2015) 100:994–1001. doi:10.1210/jc.2014-2761

Bouilly J, Beau I, Barraud S, Bernard V, Azibi K, Fagart J, Fèvre A, Todeschini AL, Veitia RA, Beldjord C, et al. Identification of Multiple Gene Mutations Accounts for a new Genetic Architecture of Primary Ovarian Insufficiency. The Journal of Clinical Endocrinology & Metabolism (2016) 101:4541–4550. doi:10.1210/jc.2016-2152

Bovolenta M, Neri M, Martoni E, Urciuolo A, Sabatelli P, Fabris M, Grumati P, Mercuri E, Bertini E, Merlini L, et al. Identification of a deep intronic mutation in the COL6A2 gene by a novel custom oligonucleotide CGH array designed to explore allelic and genetic heterogeneity in collagen VI-related myopathies. BMC Medical Genetics (2010) 11: doi:10.1186/1471-2350-11-44

Boyce AE, Mcgrath JA, Techanukul T, Murrell DF, Chow CW, Mcgregor L, Warren LJ. Ectodermal dysplasia-skin fragility syndrome due to a new homozygous internal deletion mutation in the PKP1 gene. Australasian Journal of Dermatology (2011) 53:61–65. doi:10.1111/j.1440-0960.2011.00846.x

Caburet S, Arboleda VA, Llano E, Overbeek PA, Barbero JL, Oka K, Harrison W, Vaiman D, Ben-Neriah Z, García-Tuñón I, et al. Mutant Cohesin in Premature Ovarian Failure. New England Journal of Medicine (2014) 370:943–949. doi:10.1056/nejmoa1309635

Carlosama C, Elzaiat M, Patiño LC, Mateus HE, Veitia RA, Laissue P. A homozygous donor splice-site mutation in the meiotic gene MSH4 causes primary ovarian insufficiency. Human Molecular Genetics (2017) doi:10.1093/hmg/ddx199

Carminho-Rodrigues MT, Klee P, Laurent S, Guipponi M, Abramowicz M, Cao-Van H, Guinand N, Paoloni-Giacobino A. LARS2-Perrault syndrome: a new case report and literature review. BMC Medical Genetics (2020) 21: doi:10.1186/s12881-020-01028-8

Castéra L, Krieger S, Rousselin A, Legros A, Baumann J-J, Bruet O, Brault B, Fouillet R, Goardon N, Letac O, et al. Next-generation sequencing for the diagnosis of hereditary breast and ovarian cancer using genomic capture targeting multiple candidate genes. European Journal of Human Genetics (2014) 22:1305–1313. doi:10.1038/ejhg.2014.16

Cattoni A, Spano A, Tulone A, Boneschi A, Masera N, Maitz S, Blasio AMD, Persani L, Guizzardi F, Rossetti R. The Potential Synergic Effect of a Complex Pattern of Multiple Inherited Genetic Variants as a Pathogenic Factor for Ovarian Dysgenesis: A Case Report. Frontiers in Endocrinology (2020) 11: doi:10.3389/fendo.2020.540683

Cooper GM, Stone EA, Asimenos G, et al. Distribution and intensity of constraint in mammalian genomic sequence. Genome Res. (2005) 15:901-913. doi:10.1101/gr.3577405

Cordts E, Santos M, Santos A, Mafra F, Christofolini D. FSHR polymorphisms are associated with premature ovarian insufficiency development. Fertility and Sterility (2013) 100: doi:10.1016/j.fertnstert.2013.07.1541

Cui T, He B, Kong S, Zhou C, Zhang H, Ni Z, Bao H, Qiu J, Xin Q, Reinberg D, et al. PR-Set7 deficiency limits uterine epithelial population growth hampering postnatal gland formation in mice. Cell Death & Differentiation (2017) 24:2013–2021. doi:10.1038/cdd.2017.120

Dazzo E, Fanciulli M, Serioli E, Minervini G, Pulitano P, Binelli S, Di Bonaventura C, Luisi C, Pasini E, Striano S, et al. Heterozygous Reelin Mutations Cause Autosomal-Dominant Lateral Temporal Epilepsy. The American Journal of Human Genetics (2015) 96:992–1000. doi:10.1016/j.ajhg.2015.04.020

DeBalsi KL, Longley MJ, Hoff KE, Copeland WC. Synergistic Effects of thein cisT251I and P587L Mitochondrial DNA Polymerase γ Disease Mutations. Journal of Biological Chemistry (2017) 292:4198–4209. doi:10.1074/jbc.m116.773341

Desai S, Wood-Trageser M, Matic J, Chipkin J, Jiang H, Bachelot A, Dulon J, Sala C, Barbieri C, Cocca M, et al. MCM8 and MCM9 Nucleotide Variants in Women with Primary Ovarian Insufficiency. The Journal of Clinical Endocrinology & Metabolism (2016) doi:10.1210/jc.2016-2565

Di Fonzo A, Bordoni A, Crimi M, Sara G, Bo RD, Bresolin N, Comi GP. POLG mutations in sporadic mitochondrial disorders with multiple mtDNA deletions. Human Mutation (2003) 22:498–499. doi:10.1002/humu.9203

Diggle CP, Martinez-Garay I, Molnar Z, Brinkworth MH, White E, Fowler E, Hughes R, Hayward BE, Carr IM, Watson CM, et al. A tubulin alpha 8 mouse knockout model indicates a likely role in spermatogenesis but not in brain development. Plos One (2017) 12: doi:10.1371/journal.pone.0174264

Dikic I, Elazar Z. Mechanism and medical implications of mammalian autophagy. Nature Reviews Molecular Cell Biology (2018) 19:349–364. doi:10.1038/s41580-018-0003-4

Dulhunty AF, Beard NA, Casarotto MG. Recent advances in understanding the ryanodine receptor calcium release channels and their role in calcium signalling. F1000Research (2018) 7:1851. doi:10.12688/f1000research.16434.1

Ellis NA, German J. Molecular genetics of Blooms syndrome. Human Molecular Genetics (1996) 5:1457–1463. doi:10.1093/hmg/5.supplement_1.1457

Ferrari I, Bouilly J, Beau I, Guizzardi F, Ferlin A, Pollazzon M, Salerno M, Binart N, Persani L, Rossetti R. Impaired protein stability and nuclear localization of NOBOX variants associated with premature ovarian insufficiency. Human Molecular Genetics (2016) doi:10.1093/hmg/ddw342

França M, Funari M, Nishi M, Narcizo A, Domenice S, Costa E, Lerario A, Mendonca B. Identification of the first homozygous 1-bp deletion in GDF9 gene leading to primary ovarian insufficiency by using targeted massively parallel sequencing. Clinical Genetics (2017) 93:408–411. doi:10.1111/cge.13156

França MM, Funari MFA, Lerario AM, Santos MG, Nishi MY, Domenice S, Moraes DR, Costalonga EF, Maciel GAR, Maciel-Guerra AT, Guerra-Junior G, Mendonca BB. Screening of targeted panel genes in Brazilian patients with primary ovarian insufficiency. PLoS One (2020) 23;15:e0240795. doi:10.1371/journal.pone.0240795

França MM, Funari MFA, Lerario AM, Santos MG, Nishi MY, Domenice S, Moraes DR, Costalonga EF, Maciel GAR, Maciel-Guerra AT, et al. Screening of targeted panel genes in Brazilian patients with primary ovarian insufficiency. Plos One (2020) 15: doi:10.1371/journal.pone.0240795

Gan L, Seki A, Shen K, Iyer H, Han K, Hayer A, Wollman R, Ge X, Lin JR, Dey G, et al. The lysosomal GPCR-like protein GPR137B regulates Rag and mTORC1 localization and activity. Nature Cell Biology (2019) 21:614–626. doi:10.1038/s41556-019-0321-6

Glinka A, Dolde C, Kirsch N, Huang YL, Kazanskaya O, Ingelfinger D, Boutros M, Cruciat CM, Niehrs C. LGR4 and LGR5 are R‐spondin receptors mediating Wnt/β‐catenin and Wnt/PCP signalling. EMBO reports (2011) 12:1055–1061. doi:10.1038/embor.2011.175

González-Vioque E, Blázquez A, Fernández-Moreira D, Bornstein B, Bautista J, Arpa J, Navarro C, Campos Y, Fernández-Moreno MA, Garesse R, et al. Association of Novel POLGMutations and Multiple Mitochondrial DNA Deletions With Variable Clinical Phenotypes in a Spanish Population. Archives of Neurology (2006) 63:107. doi:10.1001/archneur.63.1.107

Gostyńska KB, Nijenhuis M, Lemmink H, Pas HH, Pasmooij AM, Lang KK, Castañón MJ, Wiche G, Jonkman MF. Mutation in exon 1a of PLEC, leading to disruption of plectin isoform 1a, causes autosomal-recessive skin-only epidermolysis bullosa simplex. Human Molecular Genetics (2015) 24:3155–3162. doi:10.1093/hmg/ddv066

Grøndahl M, Andersen CY, Bogstad J, Nielsen F, Meinertz H, Borup R. Gene expression profiles of single human mature oocytes in relation to age. Human Reproduction (2010) 25:957–968. doi:10.1093/humrep/deq014

Grünblatt E, Nemoda Z, Werling AM, Roth A, Angyal N, Tarnok Z, Thomsen H, Peters T, Hinney A, Hebebrand J, et al. The involvement of the canonical Wnt-signaling receptor LRP5 and LRP6 gene variants with ADHD and sexual dimorphism: Association study and meta-analysis. American Journal of Medical Genetics Part B: Neuropsychiatric Genetics (2018) 180:365–376. doi:10.1002/ajmg.b.32695

Hu W, Feng Z. The role of p53 in reproduction, an unexpected function for a tumor suppressor. Journal of Molecular Cell Biology (2019) 11:624–627. doi:10.1093/jmcb/mjz072

Huang L, Wang H-Y, Li J-D, Wang J-H, Zhou Y, Luo R-Z, Yun J-P, Zhang Y, Jia W-H, Zheng M. KPNA2 promotes cell proliferation and tumorigenicity in epithelial ovarian carcinoma through upregulation of c-Myc and downregulation of FOXO3a. Cell Death & Disease (2013) 4: doi:10.1038/cddis.2013.256

Huang Y, Zhao M, Xu H, Wang K, Fu Z, Jiang Y, Yao Z. RASAL2 down-regulation in ovarian cancer promotes epithelial-mesenchymal transition and metastasis. Oncotarget (2014) 5:6734–6745. doi:10.18632/oncotarget.2244

Hussain MM. Structural, biochemical and signaling properties of the low-density lipoprotein receptor gene family. Frontiers in Bioscience (2001) 6:d417. doi:10.2741/hussain1

Hussein-Fikret S, Fuller P. Expression of nuclear receptor coregulators in ovarian stromal and epithelial tumours. Molecular and Cellular Endocrinology (2005) 229:149–160. doi:10.1016/j.mce.2004.08.005

Inoue T, Terada K, Furukawa A, et al. Cloning and Characterization of Mr-S, a Novel SAM Domain Protein, Predominantly Expressed in Retinal Photoreceptor Cells. BMC Developmental Biology (2006) 6:15. doi: 10.1186/1471-213X-6-15

Jiao X, Chen Z-J. Genetics of Primary Ovarian Insufficiency. eLS (2018)1–7. doi:10.1002/9780470015902.a0026636

Jin G, Long C, Liu W, et al. Identification and characterization of novel alternative splice variants of human SAMD11. Gene (2013) 530:215–221. doi: 10.1016/j.gene.2013.08.033

Kamiński A, Karasiewicz M, Bogacz A, Dziekan K, Seremak-Mrozikiewicz A, Czerny B. The importance of the Wnt/β-catenin pathwayand LRP5 protein in bone metabolism of postmenopausal women. Advances in Clinical and Experimental Medicine (2019) 28:179–184. doi:10.17219/acem/79969

Kang H-J, Rosenwaks Z. p53 and reproduction. Fertility and Sterility (2018) 109:39–43. doi:10.1016/j.fertnstert.2017.11.02

Kang KT, Kwon YW, Kim DK, Lee SI, Kim K-H, Suh D-S, Kim JH. TRRAP stimulates the tumorigenic potential of ovarian cancer stem cells. BMB Reports (2018) 51:514–519. doi:10.5483/bmbrep.2018.51.10.042

Latronico AC, Anasti J, Arnhold IJ, Rapaport R, Mendonca BB, Bloise W, Castro M, Tsigos C, Chrousos GP. Testicular and Ovarian Resistance to Luteinizing Hormone Caused by Inactivating Mutations of the Luteinizing Hormone–Receptor Gene. New England Journal of Medicine (1996) 334:507–512. doi:10.1056/nejm199602223340805

Ledig S, Röpke A, Wieacker P. Copy Number Variants in Premature Ovarian Failure and Ovarian Dysgenesis. Sexual Development (2010) 4:225–232. doi:10.1159/000314958

Li L, Zhou X, Wang X, et al (2016) A dominant negative mutation at the ATP binding domain of AMHR2 is associated with a defective anti-Müllerian hormone signaling pathway. Molecular Human Reproduction 22:669–678. doi: 10.1093/molehr/gaw040

Lin J-L, Lee W-I, Huang J-L, Chen PK-T, Chan K-C, Lo L-J, You Y-J, Shih Y-F, Tseng T-Y, Wu M-C. Immunologic assessment and KMT2D mutation detection in Kabuki syndrome. Clinical Genetics (2014) 88:255–260. doi:10.1111/cge.12484

Liu H, Wei X, Sha Y, Liu W, Gao H, Lin J, Li Y, Tang Y, Wang Y, Wang Y, et al. Whole-exome sequencing in patients with premature ovarian insufficiency: early detection and early intervention. Journal of Ovarian Research (2020) 13: doi:10.1186/s13048-020-00716-6

Luo W, Guo T, Li G, Liu R, Zhao S, Song M, Zhang L, Wang S, Chen Z-J, Qin Y. Variants in Homologous Recombination Genes EXO1 and RAD51 Related with Premature Ovarian Insufficiency. The Journal of Clinical Endocrinology & Metabolism (2020) 105: doi:10.1210/clinem/dgaa505

Luoma P, Melberg A, Rinne JO, Kaukonen JA, Nupponen NN, Chalmers RM, Oldfors A, Rautakorpi I, Peltonen L, Majamaa K et al. Parkinsonism, premature menopause, and mitochondrial DNA polymerase gamma mutations: clinical and molecular genetic study. Lancet (2017) 364:875–882. (doi:10.1016/S0140-6736(04)16983-3)

Ma J-Y, Feng X, Tian X-Y, Chen L-N, Fan X-Y, Guo L, Li S, Yin S, Luo S-M, Ou X-H. The repair of endo/exogenous DNA double-strand breaks and its effects on meiotic chromosome segregation in oocytes. Human Molecular Genetics (2019) 28:3422–3430. doi:10.1093/hmg/ddz156

Mahajan MA, Samuels HH. Nuclear receptor coactivator/coregulator NCoA6(NRC) is a pleiotropic coregulator involved in transcription, cell survival, growth and development. Nuclear Receptor Signaling (2008) 6: doi:10.1621/nrs.06002

Mathorne SW, Ravn P, Hansen D, et al. Novel phenotype of syndromic premature ovarian insufficiency associated with TP63 molecular defect. Clin Genet (2020) 97:779-784. doi:10.1111/cge.13725

Messiaen S, Bras AL, Duquenne C, Barroca V, Moison D, Déchamps N, Doussau M, Bauchet A-L, Guerquin M-J, Livera G, et al. Rad54 is required for the normal development of male and female germ cells and contributes to the maintainance of their genome integrity after genotoxic stress. Cell Death & Disease (2013) 4: doi:10.1038/cddis.2013.281

Michiels JJ, Vliet HHV. Dominant von Willebrand Disease Type 2A Groups I and II due to Missense Mutations in the A2 Domain of the von Willebrand Factor Gene: Diagnosis and Management. Acta Haematologica (2009) 121:154–166. doi:10.1159/000214856

Mohamed N-E, Hay T, Reed K, Smalley M. APC2 is Critical for Ovarian WNT Signalling Control, Fertility and Tumour Suppression. (2019) doi:10.1101/516286

Mustofa MK, Tanoue Y, Tateishi C, Vaziri C, Tateishi S. Roles of Chk2 / CHEK2 in guarding against environmentally induced DNA damage and replication‐stress. Environmental and Molecular Mutagenesis (2020) 61:730–735. doi:10.1002/em.22397

Nicole S, Chaouch A, Torbergsen T, Bauché S, Bruyckere ED, Fontenille M-J, Horn MA, Ghelue MV, Løseth S, Issop Y, et al. Agrin mutations lead to a congenital myasthenic syndrome with distal muscle weakness and atrophy. Brain (2014) 137:2429–2443. doi:10.1093/brain/awu160

Noh Y-H, Matsuda K, Hong Y-K, Kunstfeld R, Riccardi L, Koch M, Oura H, Dadras SS, Streit M, Detmar M. An N-Terminal 80 kDa Recombinant Fragment of Human Thrombospondin-2 Inhibits Vascular Endothelial Growth Factor Induced Endothelial Cell Migration In Vitro and Tumor Growth and Angiogenesis In Vivo. Journal of Investigative Dermatology (2003) 121:1536–1543. doi:10.1046/j.1523

Okutman O, Muller J, Baert Y, Serdarogullari M, Gultomruk M, Piton A, Rombaut C, Benkhalifa M, Teletin M, Skory V, et al. Exome sequencing reveals a nonsense mutation in TEX15 causing spermatogenic failure in a Turkish family. Human Molecular Genetics (2015) 24:5581–5588. doi:10.1093/hmg/ddv290

Ottolenghi C, Veitia R, Quintana-Murci L, Torchard D, Scapoli L, Souleyreau-Therville N, Beckmann J, Fellous M, Mcelreavey K. The Region on 9p Associated with 46,XY Sex Reversal Contains Several Transcripts Expressed in the Urogenital System and a Novel Doublesex-Related Domain. Genomics (2000) 64:170–178. doi:10.1006/geno.2000.6121

Owerbach D. Pro-453 to Ser mutation in CYP21 is associated with nonclassic steroid 21-hydroxylase deficiency. Molecular Endocrinology (1992) 6:1211–1215. doi:10.1210/me.6.8.1211

Pacheco S, Garcia-Caldés M, Roig I. ATR function is indispensable to allow proper mammalian follicle development. (2018) doi:10.1101/471698

Pagnamenta AT, Taanman JW, Wilson CJ, Anderson NE, Marotta R, Duncan AJ, Bitner-Glindzicz M, Taylor RW, Laskowski A, Thorburn DR et al. Dominant inheritance of premature ovarian failure associated with mutant mitochondrial DNA polymerase gamma. Human Reproduction (2006) 21:2467–2473. doi:10.1093/humrep/del076

Palmer JS, Zhao ZZ, Hoekstra C, Hayward NK, Webb PM, Whiteman DC, Martin NG, Boomsma DI, Duffy DL, Montgomery GW. Novel Variants in Growth Differentiation Factor 9 in Mothers of Dizygotic Twins. The Journal of Clinical Endocrinology & Metabolism (2006) 91:4713–4716. doi:10.1210/jc.2006-0970

Panda B, Rao L, Tosh D, Dixit H, Padmalatha V, Kanakavalli M, Raseswari T, Deenadayal M, Gupta N, Chakrabarty B, et al. Germline study ofARgene of Indian women with ovarian failure. Gynecological Endocrinology (2010) 27:572–578. doi:10.3109/09513590.2010.507282

Park J, Kwon M-S, Kim EE, et al. USP35 regulates mitotic progression by modulating the stability of Aurora B. Nature Communications (2018) doi: 10.1038/s41467-018-03107-0

Patiño L, Walton k, Mueller t, Johnson K, Stocker W, Richani D, Agapiou D, Gilchrist R, Laissue P, Harrison C. BMP15 Mutations Associated With Primary Ovarian Insufficiency Reduce Expression, Activity, or Synergy With GDF9. The Journal of Clinical Endocrinology & Metabolism (2017) doi:10.1210/jc.2016-3503

Patiño LC, Beau I, Carlosama C, Buitrago JC, González R, Suárez CF, Patarroyo MA, Delemer B, Young J, Binart N, et al. New mutations in non-syndromic primary ovarian insufficiency patients identified via whole-exome sequencing. Human Reproduction (2017) 32:1512–1520. doi:10.1093/humrep/dex089

Peixoto P, Castronovo V, Matheus N, Polese C, Peulen O, Gonzalez A, Boxus M, Verdin E, Thiry M, Dequiedt F, et al. HDAC5 is required for maintenance of pericentric heterochromatin, and controls cell-cycle progression and survival of human cancer cells. Cell Death & Differentiation (2012) 19:1239–1252. doi:10.1038/cdd.2012.3

Pérez-Casal M, Daly M, Peake A. A de novo mutation in exon 28 of the von Willebrand factor gene in a patient with type IIA von Willebrands disease coincides with an Mbol polymorphism in the von Willebrand factor pseudogene. Human Molecular Genetics (1993) 2:2159–2161. doi:10.1093/hmg/2.12.2159

Philibert P, Zenaty D, Lin L, Soskin S, Audran F, Leger J, Achermann JC, Sultan C. Mutational analysis of steroidogenic factor 1 (NR5a1) in 24 boys with bilateral anorchia: a French collaborative study. Human Reproduction (2007) 22:3255–3261. doi:10.1093/humrep/dem278

Pierce SB, Gersak K, Michaelson-Cohen R, Walsh T, Lee MK, Malach D, Klevit RE, King M-C, Levy-Lahad E. Mutations in LARS2, Encoding Mitochondrial Leucyl-tRNA Synthetase, Lead to Premature Ovarian Failure and Hearing Loss in Perrault Syndrome. The American Journal of Human Genetics (2013) 92:614–620. doi:10.1016/j.ajhg.2013.03.007

Porcu E, Cillo GM, Cipriani L, Sacilotto F, Notarangelo L, Damiano G, Dirodi M, Roncarati I. Impact of BRCA1 and BRCA2 mutations on ovarian reserve and fertility preservation outcomes in young women with breast cancer. Journal of Assisted Reproduction and Genetics (2019) 37:709–715. doi:10.1007/s10815-019-01658-9

Rogerson FM, Stanton H, East CJ, Golub SB, Tutolo L, Farmer PJ, Fosang AJ. Evidence of a novel aggrecan-degrading activity in cartilage: Studies of mice deficient in both ADAMTS-4 and ADAMTS-5. Arthritis & Rheumatism (2008) 58:1664–1673. doi:10.1002/art.23458

Roset R, Inagaki A, Hohl M, Brenet F, Lafrance-Vanasse J, Lange J, Scandura JM, Tainer JA, Keeney S, Petrini JHJ. The Rad50 hook domain regulates DNA damage signaling and tumorigenesis. Genes & Development (2014) 28:451–462. doi:10.1101/gad.236745.113

Rossetti R, Pasquale ED, Marozzi A, Bione S, Toniolo D, Grammatico P, Nelson LM, Beck-Peccoz P, Persani L. BMP15 mutations associated with primary ovarian insufficiency cause a defective production of bioactive protein. Human Mutation (2009) 30:804–810. doi:10.1002/humu.20961

Salvetti NR, Alfaro NS, Velázquez MML, Amweg AN, Matiller V, Díaz PU, Ortega HH. Alteration in localization of steroid hormone receptors and coregulatory proteins in follicles from cows with induced ovarian follicular cysts. Reproduction (2012) 144:723–735. doi:10.1530/rep-12-0188

Schimenti KJ, Feuer SK, Griffin LB, Graham NR, Bovet CA, Hartford S, Pendola J, Lessard C, Schimenti JC, Ward JO. AKAP9 Is Essential for Spermatogenesis and Sertoli Cell Maturation in Mice. Genetics (2013) 194:447–457. doi:10.1534/genetics.113.150789

Speiser PW, New MI, White PC. Molecular Genetic Analysis of Nonclassic Steroid 21-Hydroxylase Deficiency Associated with HLA-B14,DR1. New England Journal of Medicine (1988) 319:19–23. doi:10.1056/nejm198807073190104

Stolk L, Perry J, Chasman D, He C, Mangino M, Sulem P, Barbalic M, Broer L, Byrne E, Ernst F et al. Meta-analyses identify 13 novel loci associated with age at menopause and highlights DNA repair and immune pathways. Nature Genetic. (2012). 44: 260–268 doi: 10.1038/ng.1051

Strong ER, Schimenti JC. Evidence Implicating CCNB1IP1, a RING Domain-Containing Protein Required for Meiotic Crossing Over in Mice, as an E3 SUMO Ligase. Genes (2010) 1:440–451. doi:10.3390/genes1030440

Styrkarsdottir U, Thorleifsson G, Sulem P, Gudbjartsson DF, Sigurdsson A, Jonasdottir A, Jonasdottir A, Oddsson A, Helgason A, Magnusson OT, et al. Nonsense mutation in the LGR4 gene is associated with several human diseases and other traits. Nature (2013) 497:517–520. doi:10.1038/nature12124

Sun X-F, Sun X-H, Cheng S-F, Wang J-J, Feng Y-N, Zhao Y, Yin S, Hou Z-M, Shen W, Zhang X-F. Interaction of the transforming growth factor-β and Notch signaling pathways in the regulation of granulosa cell proliferation. Reproduction, Fertility and Development (2016) 28:1873. doi:10.1071/rd14398

Terauchi KJ, Shigeta Y, Iguchi T, Sato T. Role of Notch signaling in granulosa cell proliferation and polyovular follicle induction during folliculogenesis in mouse ovary. Cell and Tissue Research (2016) 365:197–208. doi:10.1007/s00441-016-2371-4

Tucker EJ, Grover SR, Robevska G, Bergen JVD, Hanna C, Sinclair AH. Identification of variants in pleiotropic genes causing “isolated” premature ovarian insufficiency: implications for medical practice. European Journal of Human Genetics (2018) 26:1319–1328. doi:10.1038/s41431-018-0140-4

Tucker EJ, Jaillard S, Grover SR, Bergen JD, Robevska G, Bell KM, Sadedin S, Hanna C, Dulon J, Touraine P, et al. TP63‐truncating variants cause isolated premature ovarian insufficiency. Human Mutation (2019) doi:10.1002/humu.23744

Wang J, Tian GG, Zheng Z, Li B, Xing Q, Wu J. Comprehensive Transcriptomic Analysis of Mouse Gonadal Development Involving Sexual Differentiation, Meiosis and Gametogenesis. Biological Procedures Online (2019) 21: doi:10.1186/s12575-019-0108-y

Waterham HR, Koster J, Romeijn GJ, Hennekam RC, Vreken P, Andersson HC, Fitzpatrick DR, Kelley RI, Wanders RJ. Mutations in the 3β-Hydroxysterol Δ24-Reductase Gene Cause Desmosterolosis, an Autosomal Recessive Disorder of Cholesterol Biosynthesis. The American Journal of Human Genetics (2001) 69:685–694. doi:10.1086/323473

Wimmer K, Beilken A, Nustede R, Ripperger T, Lamottke B, Ure B, Steinmann D, Reineke-Plaass T, Lehmann U, Zschocke J, et al. A novel germline POLE mutation causes an early onset cancer prone syndrome mimicking constitutional mismatch repair deficiency. Familial Cancer (2016) 16:67–71. doi:10.1007/s10689-016-9925-1

Wood-Trageser MA, Gurbuz F, Yatsenko SA, Jeffries EP, Kotan LD, Surti U, Ketterer DM, Matic J, Chipkin J, Jiang H, et al. MCM9 Mutations Are Associated with Ovarian Failure, Short Stature, and Chromosomal Instability. The American Journal of Human Genetics (2014) 95:754–762. doi:10.1016/j.ajhg.2014.11.002

Xiao W-J, He W-B, Zhang Y-X, Meng L-L, Lu G-X, Lin G, Tan Y-Q, Du J. In-Frame Variants in STAG3 Gene Cause Premature Ovarian Insufficiency. Frontiers in Genetics (2019) 10: doi:10.3389/fgene.2019.01016

Yang X, Zhang X, Jiao J, Zhang F, Pan Y, Wang Q, Chen Q, Cai B, Tang S, Zhou Z, et al. Rare variants in FANCA induce premature ovarian insufficiency. Human Genetics (2019) 138:1227–1236. doi:10.1007/s00439-019-02059-9

Yue Li, Führer M, Bahrami E, Socha P, Klaudel-Dreszler M, Bouzidi A, Liu Y, Lehle A, Magg T, Hollizeck S, et al. Human RIPK1 deficiency causes combined immunodeficiency and inflammatory bowel diseases. Proc Natl Acad Sci USA. (2019) 116:970-975. doi: 10.1073/pnas.1813582116.

Zegerman P. DNA Replication: Polymerase Epsilon as a Non-catalytic Converter of the Helicase. Current Biology (2013) 23: doi:10.1016/j.cub.2013.03.008

Zielen A, Khan M, Pollock N, Jiang H, Ahmed J, Nazli R, Jabeen M, Yatsenko A, Rajkovic A. A novel homozygous frame-shift variant in the LHCGR gene is associated with primary ovarian insufficiency in a Pakistani family. Clinical Genetics (2018) 94:396–397. doi:10.1111/cge.13406
