# Supplementary material for: Targeted Next-Generation Sequencing Indicates a Frequent Oligogenic Involvement in Primary Ovarian Insufficiency Onset
Source: Front Endocrinol (Lausanne). 2021 Nov 4;12:664645. doi: 10.3389/fendo.2021.664645 (PMC8600266; doi:10.3389/fendo.2021.664645)
Supplement: Supplementary file 3 [file Table_3.docx]

Supplementary Material

**Table S3. Summary of** **the variants harbored by each patient obtained after *OVO-Array* NGS panel analysis.** The phenotype of each patient is also reported. Recurrent genes are represented using the same colors.

| **N. of Variants/Patient** | **Patients ID** | **Phenotype** | **Genes found altered** |
| --- | --- | --- | --- |
| 6 | 1 | PA | \| ANAPC1 \| ATR \| LARS2 \| LARS2 \| MLH3 \| POLE \| \| --- \| --- \| --- \| --- \| --- \| --- \| |
|  | 2 | early SA | \| ATM \| COL6A2 \| RASAL2 \| RYR3 \| TP73 \| CYP21A2 \| \| --- \| --- \| --- \| --- \| --- \| --- \| |
| 5 | 3 | OD | \| ADAMTS16 \| ATM \| BLM \| CYP21A2 \| FSHR \| \| --- \| --- \| --- \| --- \| --- \| |
|  | 4 | PA | \| RAD54L \| RYR3 \| NCOR2 \| RAD52 \| MCM9 \| \| --- \| --- \| --- \| --- \| --- \| |
|  | 5 | SA | \| ERBB4 \| ERBB4 \| PKP1 \| RYR3 \| SAMD11 \| \| --- \| --- \| --- \| --- \| --- \| |
| 4 | 6 | OD | \| FIGLA \| NCOR2 \| NOBOX \| NR5A1 \| \| --- \| --- \| --- \| --- \| |
|  | 7 | PA | \| NOTCH3 \| TRRAP \| VWF \| VWF \| \| --- \| --- \| --- \| --- \| |
|  | 8 | early SA | \| SAMD11 \| ATG4C \| NOTCH4 \| RMI1 \| \| --- \| --- \| --- \| --- \| |
|  | 9 | early SA | \| NR5A1 \| SYNE1 \| RAD50 \| ADAMTS5 \| \| --- \| --- \| --- \| --- \| |
|  | 10 | SA | \| ADAMTS4 \| DHCR24 \| FSHR \| RAD52 \| \| --- \| --- \| --- \| --- \| |
|  | 11 | SA | \| APC2 \| ATG2A \| COL6A1 \| KPNA2 \| \| --- \| --- \| --- \| --- \| |
|  | 12 | SA | \| LARS2 \| RBBP8 \| THBS2 \| RIPK1 \| \| --- \| --- \| --- \| --- \| |
|  | 13 | SA | \| ATM \| NCOR2 \| POLG \| POLG \| \| --- \| --- \| --- \| --- \| |
|  | 14 | SA | \| VWF \| VWF \| LGR4 \| HK3 \| \| --- \| --- \| --- \| --- \| |
| 3 | 15 | PA | \| TEX15 \| TUBA8 \| ID1 \| \| --- \| --- \| --- \| |
|  | 16 | PA | \| ATR \| POLG \| POLG \| \| --- \| --- \| --- \| |
|  | 17 | early SA | \| NOTCH2 \| TP53 \| SAMD11 \| \| --- \| --- \| --- \| |
|  | 18 | SA | \| ERBB3 \| POLG \| LARS2 \| \| --- \| --- \| --- \| |
|  | 19 | SA | \| RAD52 \| RBBP8 \| PLEC \| \| --- \| --- \| --- \| |
|  | 20 | SA | \| ATG4C \| ATM \| PRIM1 \| \| --- \| --- \| --- \| |
|  | 21 | SA | \| SAMD11 \| KMT2D \| NBN \| \| --- \| --- \| --- \| |
|  | 22 | SA | \| COL6A2 \| APC2 \| LRP5 \| \| --- \| --- \| --- \| |
|  | 23 | SA | \| BMP15 \| GDF9 \| GDF9 \| \| --- \| --- \| --- \| |
| 2 | 24 | OD | \| NOBOX \| STAG3 \| \| --- \| --- \| |
|  | 25 | PA | \| REC8 \| LHCGR \| \| --- \| --- \| |
|  | 26 | PA | \| AGRN \| VLDLR \| \| --- \| --- \| |
|  | 27 | PA | \| TP63 \| FANCA \| \| --- \| --- \| |
|  | 28 | early SA | \| DMRT3 \| RELN \| \| --- \| --- \| |
|  | 29 | early SA | \| HDAC5 \| AKAP9 \| \| --- \| --- \| |
|  | 30 | SA | \| SAMD11 \| HK3 \| \| --- \| --- \| |
|  | 31 | SA | \| CCNB1IP1 \| MLH3 \| \| --- \| --- \| |
|  | 32 | SA | \| MSH4 \| CYP21A2 \| \| --- \| --- \| |
|  | 33 | SA | \| SAMD11 \| COL6A2 \| \| --- \| --- \| |
|  | 34 | SA | \| GPR137C \| RELN \| \| --- \| --- \| |
| 1 | 35 | PA | \| RAD54L \| \| --- \| |
|  | 36 | early SA | \| BRCA1 \| \| --- \| |
|  | 37 | early SA | \| AR \|  \| \| --- \| --- \| |
|  | 38 | early SA | \| COL6A2 \|  \| \| --- \| --- \| |
|  | 39 | SA | \| RYR3 \|  \| \| --- \| --- \| |
|  | 40 | SA | \| CHEK2 \|  \| \| --- \| --- \| |
|  | 41 | SA | \| USP35 \|  \| \| --- \| --- \| |
|  | 42 | SA | \| PKP1 \|  \| \| --- \| --- \| |
|  | 43 | SA | \| KMT5A \|  \| \| --- \| --- \| |
|  | 44 | SA | \| NR5A1 \|  \| \| --- \| --- \| |
|  | 45 | SA | \| NCOA6 \|  \| \| --- \| --- \| |
|  | 46 | SA | \| ADAMTS5 \|  \| \| --- \| --- \| |
|  | 47 | SA | \| SAMD11 \|  \| \| --- \| --- \| |
|  | 48 | SA | \| POLG \|  \| \| --- \| --- \| |

OD, Ovarian Dygenesis; PA, Primary Amenorrhea; SA, Secondary Amenorrhea; early SA, only menarche or spotting episodes.
