# Supplementary material for: Targeted Next-Generation Sequencing Indicates a Frequent Oligogenic Involvement in Primary Ovarian Insufficiency Onset
Source: Front Endocrinol (Lausanne). 2021 Nov 4;12:664645. doi: 10.3389/fendo.2021.664645 (PMC8600266; doi:10.3389/fendo.2021.664645)
Supplement: Supplementary file 4 [file Table_4.docx]

Supplementary Material

**Table S4. Summary of the candidate gene variations and *in-silico* predictions found in the cohort of 43 patients screened for diagnostic routine in known POI genes.** Frequencies of the variants in the POI group and in the female population are reported for each gene variant, together with VarSome prediction. For table indicators, please refer to Table S2.

| **Gene** | **Transcript** | **cDNA Variation** | **Protein Variation** | **Diagnostic POI cases frequency (n=43)** | **gnomAD ver. 2.1.1 female population frequency** | **VarSome Pathogenicity Scores** | **Link to VarSome** | **References** |
| --- | --- | --- | --- | --- | --- | --- | --- | --- |
| *BMP15* | NM_005448 | c.202C>T | p.Arg68Trp | 0.0232558 | 0.000708 | 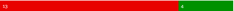 | varso.me/Ne2w | Rossetti *et al.*, 2009 |
|  |  | c.406G>C | p.Val136Leu | 0.0232558 | 0.00000866 | 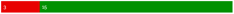 | varso.me/TZn5 |  |
| *FIGLA* | - | - | - | - | - | - | - |  |
| *FOXL2* | - | - | - | - | - | - | - |  |
| *FSHR* | NM_000145 | c.926G>C | p.Gly309Ala | 0.0465116 | Novel | 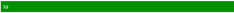 | varso.me/S1eC |  |
|  |  | c.1964G>C | p.Arg655Thr | 0.0232558 | Novel | 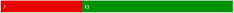 | varso.me/S1eT |  |
|  |  | c.909-917del | p.Tyr303* | 0.0232558 | 0.00000867 | GERP NR 5.441 (m.n.)  GERP RS 3.4941 (m.n.) | varso.me/UO5b |  |
|  |  | c.1118G>A | p.Ser373Asn | 0.0232558 | - | 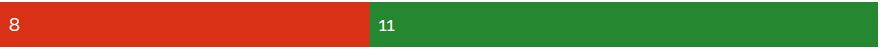 | varso.me/TZcz |  |
|  |  | c.394A>T | p.Ile132Phe | 0.0232558 | Novel | 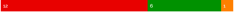 | varso.me/T1ug |  |
| GDF9 | NM_005260 | c.362C>T | p.Thr121Ile | 0.0232558 | 0.000277 | 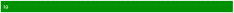 | varso.me/TZ8s | Bouilly *et al.*, 2016; Palmer *et al.*, 2006 |
|  |  | c.566C>T | p.Thr189Ile | 0.0232558 | Novel | 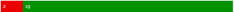 | varso.me/TZ8M |  |
|  |  | c.307C>T | p.Pro103Ser | 0.0232558 | 0.0028 | 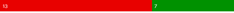 | varso.me/TZ99 |  |
| *NOBOX* | NM_001080413 | c.331G>A | p.Gly111Arg | 0.0232558 | 0.0000131 | 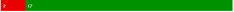 | varso.me/TZr6 |  |
| *NR5A1* | - | - | - | - | - | - | - |  |
| *SYCE1* | - | - | - | - | - | - | - |  |
| *STAG3* | NM_012447 | c.3433G>A | p.Glu1145Lys | 0.0232558 | 0.0000346 | 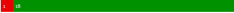 | varso.me/S1e5 |  |
|  |  | c.1678-10_2228del | p.? | 0.0232558 | Novel | GERP NR 5.1175 (m.n.)  GERP RS 0.5749 (m.n.) | varso.me/TZuL |  |

**References**

Bouilly J, Beau I, Barraud S, Bernard V, Azibi K, Fagart J, Fèvre A, Todeschini AL, Veitia RA, Beldjord C, et al. Identification of Multiple Gene Mutations Accounts for a new Genetic Architecture of Primary Ovarian Insufficiency. The Journal of Clinical Endocrinology & Metabolism (2016) 101:4541–4550. doi:10.1210/jc.2016-2152

Palmer JS, Zhao ZZ, Hoekstra C, Hayward NK, Webb PM, Whiteman DC, Martin NG, Boomsma DI, Duffy DL, Montgomery GW. Novel Variants in Growth Differentiation Factor 9 in Mothers of Dizygotic Twins. The Journal of Clinical Endocrinology & Metabolism (2006) 91:4713–4716. doi:10.1210/jc.2006-0970

Rossetti R, Pasquale ED, Marozzi A, Bione S, Toniolo D, Grammatico P, Nelson LM, Beck-Peccoz P, Persani L. BMP15 mutations associated with primary ovarian insufficiency cause a defective production of bioactive protein. Human Mutation (2009) 30:804–810. doi:10.1002/humu.20961
