# Supplementary material for: Targeted Next-Generation Sequencing Indicates a Frequent Oligogenic Involvement in Primary Ovarian Insufficiency Onset
Source: Front Endocrinol (Lausanne). 2021 Nov 4;12:664645. doi: 10.3389/fendo.2021.664645 (PMC8600266; doi:10.3389/fendo.2021.664645)
Supplement: Supplementary file 5 [file Table_5.docx]

Supplementary Material

**Table S5. Summary of the variants harbored by each patient obtained by NGS analysis on known POI gene for diagnostic routine.** The phenotype of each patient is also reported. Recurrent genes are represented using the same colors.

| **N. of Variants/Patient** | **Patients ID** | **Phenotype** | **Genes found altered** |
| --- | --- | --- | --- |
| 3 | 49 | SA | \| FSHR \| FSHR \| STAG3 \| \| --- \| --- \| --- \| |
| 2 | 50 | SA | \| FSHR \| FSHR \| \| --- \| --- \| |
| 1 | 51 | ~~PA with~~ OD | \| FSHR \| \| --- \| |
|  | 52 | PA | \| GDF9 \|  \| \| --- \| --- \| |
|  | 53 | PA | \| GDF9 \|  \| \| --- \| --- \| |
|  | 54 | PA | \| BMP15 \|  \| \| --- \| --- \| |
|  | 55 | PA | \| BMP15 \|  \| \| --- \| --- \| |
|  | 56 | PA | \| FSHR \| \| --- \| |
|  | 57 | PA | \| GDF9 \|  \| \| --- \| --- \| |
|  | 58 | PA | \| STAG3 \| \| --- \| |
|  | 59 | SA | \| NOBOX \|  \| \| --- \| --- \| |

OD, Ovarian Dygenesis; PA, Primary Amenorrhea; SA, Secondary Amenorrhea; early SA, only menarche or spotting episodes.
