# Supplementary material for: Targeted Next-Generation Sequencing Indicates a Frequent Oligogenic Involvement in Primary Ovarian Insufficiency Onset
Source: Front Endocrinol (Lausanne). 2021 Nov 4;12:664645. doi: 10.3389/fendo.2021.664645 (PMC8600266; doi:10.3389/fendo.2021.664645)
Supplement: Supplementary file 6 [file Table_6.docx]

Supplementary Material

**Table S6. The following table shows the most relevant pathways identified by Reactome analysis and sorted by p-values corrected for False Discovery Rates using the Benjamini-Hochberg method. Reactome reports also “Reactions” as pathway steps defined as any biologic event that changes the state of a molecule (e.g. binding, activation, translocation, degradation).**

|  | **Entities** | | | | |  | **Reactions** | |
| --- | --- | --- | --- | --- | --- | --- | --- | --- |
| **Pathway name** | **found** | **genes** | **ratio** | **p-value** | **FDR*** |  | **found** | **ratio** |
| **Cell Cycle Checkpoints** | 14/251 | *ANAPC1, ATR, CHEK2, P53, RMI1, APC2, BLM, NBN, RAD50, ATM, BRCA1, NOTCH3, RBBP8* | 0.025 | 2.27E-04 | 1.10E-02 |  | 40/56 | 0.004 |
| **G2/M DNA damage checkpoint** | 11/113 | *ATM, BRCA1, P53, RMI1, ATR, CHEK2, RAD50, BLM, NBN, RBBP8* | 0.005 | 2.52E-08 | 2.82E-05 |  | 9/12 | 9.15E-04 |
| **Regulation of TP53 Activity through Phosphorylation** | 13/205 | *ATM, BRCA1, P53, RMI1, ATR, CHEK2, RAD50, BLM, NBN, RBBP8* | 0.01 | 7.15E-07 | 4.00E-04 |  | 25/26 | 0.002 |
| **TP53 Regulates Transcription of DNA Repair Genes** | 12/200 | *ATM, BRCA1, ATR, P53* | 0.01 | 4.29E-06 | 7.98E-04 |  | 15/17 | 0.001 |
| **Meiosis** | 20/546 | *ATM, BRCA1, MSH4, RAD50, STAG3, ATR, MLH3, MSH5, RBBP8, SYNE1, BLM, MND1, NBN, REC8, TEX15* | 0.026 | 2.52E-05 | 0.002 |  | 12/15 | 0.001 |
| **Meiotic recombination** | 15/293 | *ATM, MLH3, MSH5, RBBP8, BLM, MND1, NBN, TEX15, BRCA1, MSH4, RAD50* | 0.014 | 2.04E-05 | 0.002 |  | 6/9 | 6.86E-04 |
| **Homologous DNA Pairing and Strand Exchange** | 11/144 | *ATM, BRCA1, RAD50, ATR, NBN, RBBP8, BLM, PLEC, RMI1* | 0.007 | 2.22E-06 | 7.98E-04 |  | 7/8 | 6.10E-04 |
| **Presynaptic phase of homologous DNA pairing and strand exchange** | 10/136 | *ATM, BRCA1, RBBP8, ATR, NBN, RMI1, BLM, RAD50* | 0.006 | 1.27E-05 | 0.002 |  | 5/5 | 3.81E-04 |
| **Resolution of D-Loop Structures** | 10/175 | *ATM, NBN, RBBP8, BLM, PLEC, RMI1, BRCA1, RAD50* | 0.008 | 7.41E-05 | 0.005 |  | 8/10 | 7.63E-04 |
| **HDR through Homologous Recombination (HRR)** | 13/283 | *ATM, BRCA1, POLE, RMI1, ATR, NBN, RAD50, BLM, PLEC, RBBP8* | 0.013 | 8.02E-05 | 0.005 |  | 16/19 | 1.00E-03 |
| **HDR through Single Strand Annealing (SSA)** | 12/231 | *ATM, BRCA1, RAD52, ATR, NBN, RBBP8, BLM, RAD50, RMI1* | 0.011 | 8.85E-05 | 0.005 |  | 6/6 | 4.58E-04 |
| **Diseases of glycosylation** | 9/214 | *ADAMTS16, AGRN, NOTCH3, ADAMTS4, GLI2, NOTCH4, ADAMTS5, NOTCH2, THBS2* | 0.01 | 4.97E-05 | 0.004 |  | 15/77 | 6.00E-03 |
| **Diseases associated with O-glycosylation of proteins** | 7/80 | *ADAMTS16, ADAMTS4, ADAMTS5, NOTCH2, NOTCH3, NOTCH4, THBS2* | 0.004 | 3.56E-06 | 7.98E-04 |  | 2/9 | 6.86E-04 |
| **O-glycosylation of TSR domain containing proteins** | 4/41 | *ADAMTS16, ADAMTS4, ADAMTS5, THBS2* | 0.002 | 3.15E-04 | 0.014 |  | 2/2 | 1.53E-04 |
| **Pre-NOTCH Expression and Processing** | 12/283 | *NOTCH2, NOTCH4, NOTCH3, P53* | 0.013 | 8.02E-05 | 0.005 |  | 31/38 | 0.003 |
| **Pre-NOTCH Processing in the Endoplasmic Reticulum** | 3/16 | *NOTCH2, NOTCH3, NOTCH4* | 7.62E-04 | 2.82E-04 | 0.012 |  | 2/2 | 1.53E-04 |
| **Defective LFNG (lunatic fringe; MIM:602576)** | 3/6 | *NOTCH2, NOTCH3, NOTCH4* | 2.86E-04 | 1.57E-05 | 0.002 |  | 1/1 | 7.63E-05 |
| **ERBB2 Regulates Cell Motility** | 4/38 | *ERBB3, ERBB4* | 0.002 | 2.37E-04 | 0.011 |  | 2/2 | 1.53E-04 |
| **ERBB2 Activates PTK6 Signaling** | 4/46 | *ERBB3, ERBB4* | 0.002 | 4.85E-04 | 0.019 |  | 2/2 | 1.53E-04 |
| **Reproduction** | 20/624 | *ATM, BRCA1, MSH4, RAD50, STAG3, ATR, MLH3, MSH5, RBBP8, SYNE1, BLM, MND1, NBN, REC8, TEX15* | 0.03 | 1.12E-04 | 0.006 |  | 12/24 | 0.002 |

* False Discovery Rate
