# Supplementary material for: Targeted Next-Generation Sequencing Indicates a Frequent Oligogenic Involvement in Primary Ovarian Insufficiency Onset
Source: Front Endocrinol (Lausanne). 2021 Nov 4;12:664645. doi: 10.3389/fendo.2021.664645 (PMC8600266; doi:10.3389/fendo.2021.664645)
Supplement: Supplementary file 7 [file Table_7.docx]

Supplementary Material

**Table S7. The following table shows the most relevant pathways identified by David analysis and sorted by p-value corrected for False Discovery Rates using the Benjamini-Hochberg method.**

| **Pathway name** | **Genes** | **Count** | **p-value** | **FDR*** |
| --- | --- | --- | --- | --- |
| DNA repair | *ATM, ATR, BRCA1, BLM, POLE, FANCA, RAD50, RAD52, RAD54L, RBBP8, NBN, NCOA6, TEX15, TRRAP* | 14 | 4.0E-11 | 1.6E-8 |
| [DNA replication](http://www.ebi.ac.uk/QuickGO/GTerm?id=GO:0006260) | *ATM, ATR, BRCA1, BLM, POLE, POLG, RAD50, RBBP8, RMI1, MCM9, NBN, NCOA6* | 12 | 9.9E-11 | 2.6E-8 |
| [DNA damage](http://www.uniprot.org/keywords/?query=DNA%20damage) | *ATM, ATR, BRCA1, POLE, FANCA, RAD50, RAD52, RAD54L, RBBP8, CHEK2, MCM9, MLH3, NBN, TEX15* | 14 | 7.0E-10 | 6.7E-8 |
| [Cell cycle](http://www.uniprot.org/keywords/?query=Cell%20cycle) | *ATM, BRCA1, RAD50, RBBP8, ANAPC1, CHEK2, KMT5A, NBN, STAG3,TP53, TP63, TP73* | 12 | 3.3E-5 | 6.4E-4 |
| [Double-strand break repair via homologous recombination](http://www.ebi.ac.uk/QuickGO/GTerm?id=GO:0000724) | *ATM, BRCA1, BLM, RAD50, RAD54L, RBBP8, REC8, MCM9, NBN* | 9 | 1.4E-9 | 2.0E-7 |
| [Regulation of signal transduction by p53 class mediator](http://www.ebi.ac.uk/QuickGO/GTerm?id=GO:1901796) | *ATM, ATR, BRCA1, BLM, RAD50, RBBP8, RMI1, CHEK2, KMT5A, NBN, TP53, TP63, TP73* | 13 | 3.2E-13 | 2.5E-10 |
| [Meiosis](http://www.uniprot.org/keywords/?query=Meiosis) | *RAD50, RBBP8, REC8, CCNB1IP1, MSH4,NBN, STAG3, TEX15* | 8 | 1.60E-08 | 9.60E-06 |
| [Reciprocal meiotic recombination](http://www.ebi.ac.uk/QuickGO/GTerm?id=GO:0007131) | *ATM, RAD50, REC8, CCNB1IP1, MLH3, MSH4* | 6 | 2.20E-07 | 1.90E-05 |
| [Female gamete generation](http://www.ebi.ac.uk/QuickGO/GTerm?id=GO:0007292) | *BMP15, FSHR, GDF9, MCM9, MSH4* | 5 | 6.80E-07 | 4.90E-05 |
| Premature ovarian failure | *NOBOX, BMP15, FIGLA, NR5A1, STAG3* | 5 | 9.20E-08 | 3.60E-06 |
| Ovarian steroidogenesis | *BMP15, FSHR, GDF9, LHCGR* | 4 | 6.50E-03 | 8.20E-02 |
| [Male genitalia development](http://www.ebi.ac.uk/QuickGO/GTerm?id=GO:0030539) | *DHCR24, LGR4, LHCGR, TEX15* | 4 | 6.90E-05 | 3.10E-03 |
| [Spermatogenesis](http://www.ebi.ac.uk/QuickGO/GTerm?id=GO:0007283) | *REC8, AR, FSHR, LGR4, MSH4, TEX15, TP63* | 7 | 7.90E-03 | 1.50E-01 |
| [Extracellular matrix](http://www.uniprot.org/keywords/?query=Extracellular%20matrix) | *ADAMTS16, ADAMTS4, ADAMTS5, AGRN, COL6A1, COL6A2, RELN, VWF* | 8 | 5.6E-5 | 8.2E-4 |
| [ECM-receptor interaction](http://www.ebi.ac.uk/QuickGO/GTerm?id=GO:0030198) | *AGRN, COL6A1, COL6A2, RELN, THBS2, VWF* | 6 | 5.60E-04 | 2.50E-02 |
| Notch signaling pathway | *NOTCH2, NOTCH3, NOTCH4, NCOR2* | 4 | 6.20E-03 | 8.20E-02 |

* False Discovery Rate
